# Cohesive Living Bacterial Films with Tunable Mechanical Properties from Cell Surface Protein Display

**DOI:** 10.1021/acssynbio.4c00528

**Published:** 2024-11-01

**Authors:** Hanwei Liu, Priya K. Chittur, Julia A. Kornfield, David A. Tirrell

**Affiliations:** Division of Chemistry and Chemical Engineering, California Institute of Technology, Pasadena, California 91125, United States

**Keywords:** engineered living materials, protein surface display, tunable mechanical properties, self-healing materials, disulfide engineering

## Abstract

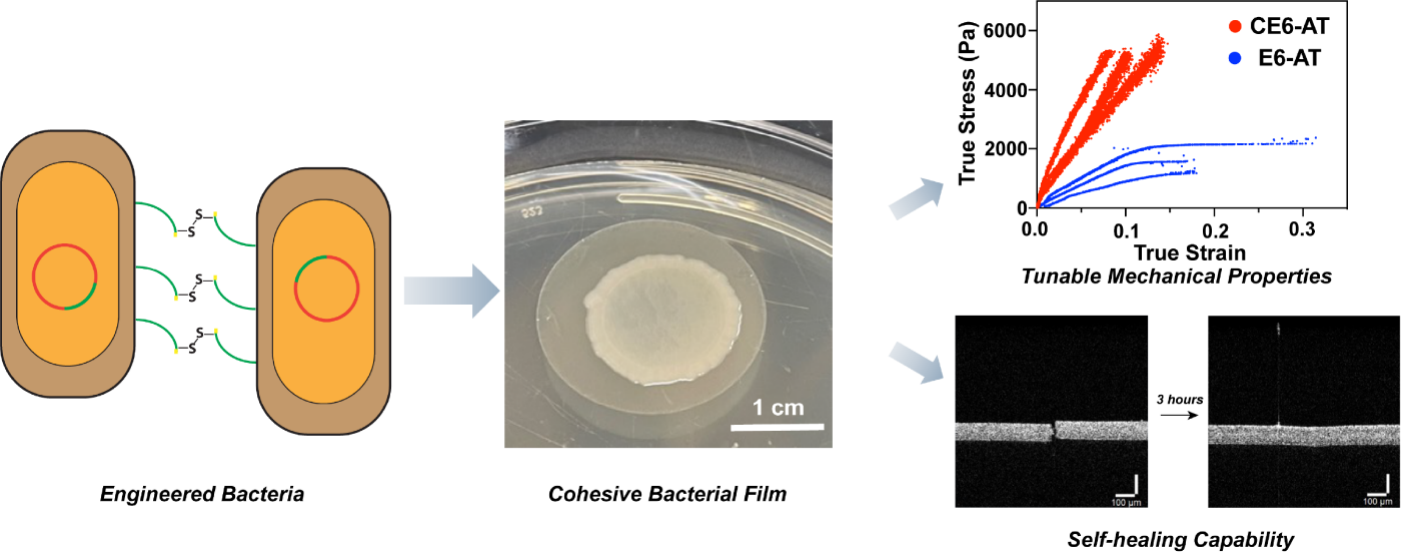

Engineered living materials (ELMs) constitute a novel class of functional materials that contain living organisms. The mechanical properties of many such systems are dominated by the polymeric matrices used to encapsulate the cellular components of the material, making it hard to tune the mechanical behavior through genetic manipulation. To address this issue, we have developed living materials in which mechanical properties are controlled by the cell-surface display of engineered proteins. Here, we show that engineered *Esherichia coli* cells outfitted with surface-displayed elastin-like proteins (ELPs, designated E6) grow into soft, cohesive bacterial films with biaxial moduli around 14 kPa. When subjected to bulge-testing, such films yielded at strains of approximately 10%. Introduction of a single cysteine residue near the exposed N-terminus of the ELP (to afford a protein designated CE6) increases the film modulus 3-fold to 44 kPa and eliminates the yielding behavior. When subjected to oscillatory stress, films prepared from *E. coli* strains bearing CE6 exhibit modest hysteresis and full strain recovery; in E6 films much more significant hysteresis and substantial plastic deformation are observed. CE6 films heal autonomously after damage, with the biaxial modulus fully restored after a few hours. This work establishes an approach to living materials with genetically programmable mechanical properties and a capacity for self-healing. Such materials may find application in biomanufacturing, biosensing, and bioremediation.

## Introduction

In nature, microorganisms including bacteria can autonomously assemble into hierarchical biofilms,^1^ composed in part of living cells that can sense environmental stress^2^ and catalyze reactions,^3,4^ and in part of extracellular polymeric matrices (EPS)^5^ that are secreted by cells and composed of proteins, lipids, polysaccharides, and nucleic acids.^6^ These polymeric matrices create microenvironments that help bacteria survive environmental challenges.^7^ Inspired by natural biofilms and other biomaterials, the field of engineered living materials lies at the interface between materials science and synthetic biology. Through manipulation of genetic information, organisms can be directed to assemble into materials that possess desirable characteristics of living systems, such as autonomous assembly, adaptiveness to environmental stimuli, and self-healing.^8−12^ Recently, several biofilm-inspired living materials have been reported, in which researchers either rewired the production of natural biopolymers like curli fibrils^13,14^ or cellulose,^15,16^ or encapsulated bacteria in synthetic polymeric matrices.^17,18^ Proteins and peptides have been fused to curli protein monomers and assembled into materials that exhibit novel catalytic functions.^19,20^ Cellulose-producing bacteria assembled at the air–water interface^21,22^ form pellicles that can be collected and processed via 3D printing.^15^ Encapsulating bacteria in synthetic polymer networks can protect the cells from environmental insults while preserving sensitivity to environmental stimuli.^18^ In such materials, the polymeric matrix dominates the mechanical properties, while the encapsulated cells serve as environmental sensors.

Matrix-free living materials have also been described. In such systems, bacteria are engineered to express associative proteins and display them on the cell surface, forming cell aggregates. This approach requires neither matrix secretion nor synthetic polymers, so that living materials, in principle, can be generated from a single cell. Materials of this kind have been developed on the basis of nanobody-antigen interactions in *E. coli*,^23,24^ and through engineering *Caulobacter crescentus* by modifying its RsaA protein.^25,26^ In prior examples, cells were grown in liquid culture and then collected and assembled into cm-scale structures. However, the growth of centimeter-scale living cohesive bacterial films with tunable mechanical properties on solid surfaces, utilizing surface-displayed self-associative proteins, has not been reported.

Here, we explore the possibility of *growing* a cohesive bacterial film directly on a solid substrate using a previously developed protein surface-display system^27^ to engineer *E. coli* to display elastin-like proteins, with the introduction of a single amino acid that enables covalent links between neighboring cells. Surface display of unstructured elastin-like proteins, 150 amino acids in length, enabled bacterial cells to form cohesive films. The introduction of a single cysteine residue near the exposed N-terminus of the displayed elastin improved the mechanical performance of the films, presumably by forming intercellular disulfide bonds. The resulting films without the cysteine residue yield when nominal stress exceeds 1 to 2 kPa; in contrast, films with the cysteine did not undergo yielding even when subjected to nominal stress of 4 to 5 kPa, survived multiple loading cycles without permanent deformation, and exhibited self-healing after fracture, with some recovering their moduli fully within a few hours.

## Results and Discussion

### Design of Surface-Displayed Proteins

**Figure 1:**
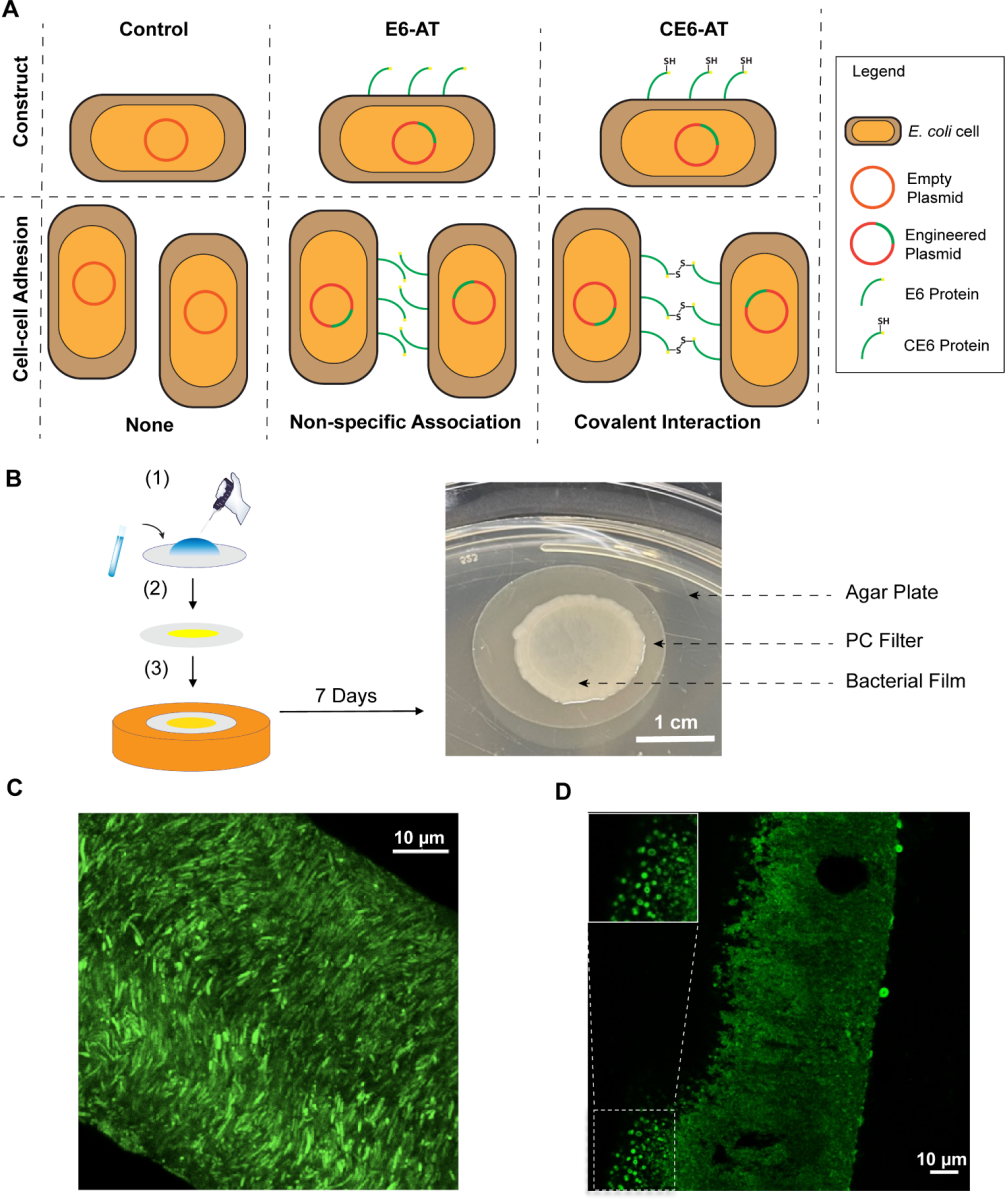
Cohesive living films made from engineered bacteria. (A) Schematic representations of engineered bacteria and types of intercellular adhesion. (B) Suction-coating method for preparation of bacterial films. (1) Overnight planktonic culture of bacteria of interest was pipetted onto a perforated polycarbonate filter with a 0.2 μm diameter pore size. (2) Vacuum filtration was applied to remove liquid media and retain bacteria on the filter. (3) The filter with bacterial coating was then transferred to a fresh LB agar plate daily for 7 days. An image of a 7-day-old CE6-AT bacterial film on a polycarbonate filter on an LB agar plate. Scale bar: 1 cm. (C) Microscopy image of a microtomed cross-section of a 7-day-old CE6-AT film that expresses mWasabi as a fluorescence marker. Scale bar: 10 μm. (D) Microscopy image of a 7-day-old CE6-AT film microtome cross-section immunostained by an anti-His-tag antibody conjugated to Dylight-488. Scale bar, 10 μm. The image shown in the upper left corner is an enlargement of the dashed-square portion of the image in the lower left corner.

We expressed proteins on the *E. coli* surface using an autodisplay system previously used to display a wide variety of proteins, including enzymes and vaccine epitopes,^28−31^ as well as proteins that drive the formation of bacterial aggregates in planktonic culture.^27^ In these cellular constructs, the protein to be displayed is inserted between a PelB secretion peptide and the autotransporter (AT) outer membrane protein (Figure S1). Both of the displayed proteins employed in this work carry 6xHis tags and elastin-like (E) peptide domains previously used to prepare protein-based hydrogels^32−34^ and to engineer microbial assembly in planktonic culture.^27^ Because we used six copies of a 25-amino acid E domain, which had proven effective in our earlier work in planktonic systems, we refer to this construct as E6-AT (Figure 1A, center). The CE6-AT construct differs from E6-AT by a single amino acid—a cysteine (C) residue placed between the 6xHis tag and the E6 domain (Figure 1A, right). The goal of introducing cysteine near the N-terminus of the displayed protein was to enable the formation of intercellular covalent disulfide bonds. These surface-displayed proteins (sequences given in Table S2) were encoded into a pQE80 plasmid backbone under the control of a T5-lac promoter. In the *E. coli* DH10B cells used in this work, the T5-lac promoter drives constitutive expression of the surface-displayed protein; no inducer is required.

### Protocol for Growing Bacterial Films

A 200-μL volume containing approximately 10^8^ cells grown from a single colony on an agar plate was pipetted onto a 2.5 cm diameter polycarbonate membrane filter with 0.2 μm pore size, covering a circular area of roughly 90 mm^2^ (Figure 1B, left). Vacuum filtration was applied to remove the liquid medium and retain the bacterial cells on the membrane filter. The filter was transferred to an LB agar plate, which provides nutrition for bacterial growth and antibiotics to maintain the expression of the plasmid. Nutrition was replenished by transferring the filter to a fresh LB agar plate every day. Bacterial films were characterized after 7 days of growth.

### Structure of Bacterial Films

To probe the structure of bacterial films, we introduced a second plasmid to drive the constitutive expression of a fluorescent protein. An image of a microtomed cross-section of a CE6-AT film that expresses mWasabi shows that 7 days of growth (described above) provides films approximately 70 μm thick that contain densely packed bacterial cells (Figure 1C). To ensure that the elastin-like proteins were in fact displayed at the bacterial surface in these films, we stained microtomed sections with the anti-6xHis antibody conjugated to Dylight-488. Staining by fluorescent antibodies is evident across the full thickness, indicating the expression of CE6-AT protein throughout the film (Figure 1D). At higher magnification (Figure 1D, lower left), we see evidence of antibody staining surrounding the cell body and appearing to form a ring when the cell is viewed on-axis. These images suggest that His-tagged protein is expressed at the cell surface, consistent with the expected surface display of CE6-AT. Similar cross-sectional imaging was applied to E6-AT films, and flow cytometry was used to analyze cell suspensions derived from empty-vector controls and E6-AT films (Figures S2 and S3). Empty-vector control films were too fragile to allow microtome-sectioning, staining, and imaging, but flow cytometry confirms that antibody-staining of E6-AT cells is roughly 2 orders of magnitude stronger than that of controls. Taken together, these results confirm that E6-AT and CE6-AT films consist of densely packed cells with elastin-like proteins displayed at the cell surface and expressed throughout the depth of the film.

### Erosion Assay of Film Cohesion

As a first test of film cohesion, we applied a simple erosion assay. Seven-day-old bacterial films were immersed in 7 mL of PBS buffer in six-well plates and placed on a rocking platform using a 15° tilt angle at 15 turns per min. Optical densities (OD_600_) of buffers in contact with bacterial films were measured at 1 and 24 h. After 1 h of erosion in PBS, control films without surface protein expression reached an OD_600_ value above 0.1, in contrast to OD_600_ < 0.001 for both E6-AT and CE6-AT. After 24 h of erosion, the supernatant OD_600_ reached values greater than 0.3 for the control, and no film was visible. In contrast, E6-AT and CE6-AT films remained intact, and the supernatant OD_600_ remained below 0.01 (Figure S4). These results confirmed the role of surface-displayed proteins in bacterial film cohesion.

### Determination of Small-Strain Biaxial Modulus

**Figure 2:**
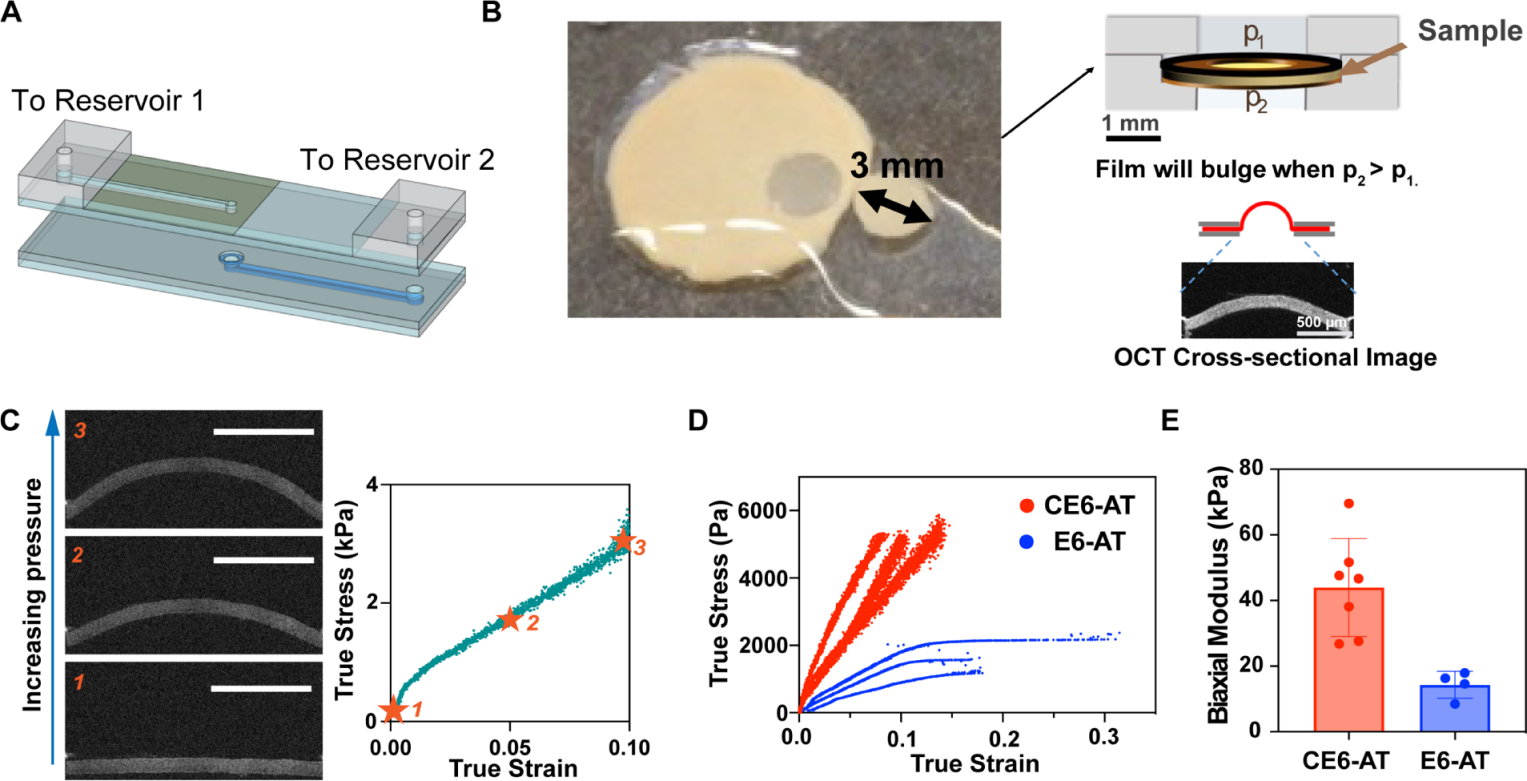
Mechanical properties of engineered bacterial films measured by the ramp bulge test. (A) Schematic of the bulge test device, fabricated as two parts that are separated to load a sample in the chamber at the center of the device; the top and bottom parts seal with vacuum grease. When loaded and sealed, “port to reservoir 1” connects to a reservoir of fluid that is used to control pressure on the top face of the sample (not shown), and “port to reservoir 2” permits control of the pressure on the bottom face of the sample (Figure S5). Gray layers are acrylic; blue indicates etched channels (light blue channel to the top face of the sample; darker blue channel to the bottom face of the sample); green shaded area indicates coverslip glass; layers are bonded using epoxy. Horizontal channels are longer than shown. (B) Schematic of loading the bacterial film sample into the bulge test device and imaging of the deformed film by optical coherence tomography (OCT). The punched bacterial film sample (3 mm diameter) supported by two washer-shaped disks was transferred to the central chamber of the device. A thin O-ring sealed this “sandwich” to the top half of the device. When pressure in reservoir 2 was larger than that in reservoir 1, a net pressure upward was applied to the bacterial film; the film bulged upward and was imaged by OCT. (C) Three representative cross-sectional OCT images (right, numbered) acquired during the bulge test of a CE6AT film under linearly increasing pressure, at the points indicated on the stress–strain curve (Right). Numbers with stars indicate data points corresponding to the images. Scale bar 500 μm. (D) Stress versus strain curves of CE6-AT and E6-AT films. Number of curves shown for CE6-AT films: 3. Number of curves shown for E6-AT films: 3. (E) Biaxial modulus values of CE6-AT and E6-AT films. Number of replicates for CE6-AT: 7. Number of replicates for E6-AT films: 4. Error bars represent 1 standard deviation.

To characterize the mechanical properties of the cohesive bacterial films, we constructed a custom millifluidic device suitable for applying a “bulge test” to the films (Figure 2A). The device imposed hydrostatic pressure differences in the Pa–kPa range across thin, freely suspended film samples, and was equipped with an optical coherence tomography (OCT) system to quantify changes in film shape, which were analyzed to extract mechanical properties.^35^ A 3-mm diameter sample, cut from a 7-day-old bacterial film, was gently covered with a drop of buffer to relieve capillary forces, lifted on a 3 mm-diameter transmission electron microscopy (TEM) disk having a 1.5 mm diameter aperture, and placed into the central chamber of the bulge test device, where another 3 mm disk with a 1.5 mm diameter aperture was placed on it (Figure 2B). Channels in the device allowed for the independent modulation of the hydrostatic pressure applied to the top and bottom faces of the film, via changes in fluid levels in two external reservoirs, “Reservoir 1” and “Reservoir 2” (Figures 2A and S5), with associated hydrostatic pressures *p*_1_ and *p*_2_. Thus, when the liquid level in “Reservoir 2” was higher than that in “Reservoir 1” (*p*_2_ > *p*_1_), the sample bulged upward (Figures 2B and S5).

To increase the pressure difference p_2_ – p_1_ at a constant rate, a syringe pump was used to add liquid to Reservoir 2 at a constant rate, imposing a “ramp” pressure–time profile. In the present experiments, PBS buffer or HEPES buffer was added at a flow rate of 70 mL/min (with 60 mL syringes used as reservoirs, the resulting rate of pressure increase was 20.4 Pa/s; see Figure S5 for additional details) until the total volume transferred reached 55 mL. The flow rate was chosen to be the maximum accommodated by the syringe pump to minimize perfusion through the biofilm for the duration of the experiment. Simultaneously, OCT images were collected (with an acquisition time of 19 ms/image). The images were subsequently processed using a custom script to extract the evolution of film stress and strain during the test (Figure 4C, detailed in the Materials and Methods section and Supporting Information).

Three stress–strain curves for CE6-AT films and three curves for E6-AT films (each curve representing a biological replicate) show an approximately linear relationship between stress and strain for CE6-AT films and a distinct yielding behavior for E6-AT. All E6-AT films were tested until failure, while none of the CE6-AT films failed even at a maximum applied pressure of 960 Pa. We also examined empty-vector control films, which were not engineered for protein surface display; those films were not cohesive and could not be loaded into the test apparatus. Biaxial moduli were estimated from the initial linear portion of each curve (below 5% strain) and were higher for CE6 than for E6, as evident in the steeper slopes of their stress–strain curves. The average biaxial modulus of CE6-AT films was determined to be 44.0 ± 5.6 kPa, approximately three times that of E6-AT films (14 ± 2.1 kPa). E6-AT films also typically yielded and transitioned to a plastic response, eventually failing before the end of the test, while CE6 films showed no evidence of yielding within the pressure regimes that we were able to test. The differences in the yielding behavior of E6-AT films and CE6-AT films were also observed when we tried to peel films from their membrane supports using tweezers without providing an aqueous medium to wet the newly exposed surfaces; E6-AT films were observed to stretch and break, while CE6-AT films could be peeled from the filter intact. Observation of CE6 failure was rare, and in our hands only occurred once (for a film that was accidentally left in 1× PBS buffer for 4 h, Figure S6). Unlike the E6 films that yielded, the one CE6 film that failed exhibited brittle failure (Figure S6). Videos of OCT-scanned “Ramp” bulge tests of E6-AT and CE6-AT films, peeling experiments, and the only observed failure event of a CE6-AT film are attached in Videos S1–S5; video frame rates are rates of actual acquisition.

### Mechanical Strength of CE6-AT Films Can Be Reduced Chemically

Relative to E6-AT films, CE6-AT films were stronger and stiffer (Figure 2D,E), supporting our hypothesis that covalent disulfide bonds between cells enhance the mechanical properties. To assess the potential role of factors other than intercellular covalent bonds in enhancing the mechanical properties of CE6-AT films, the water content (Figure S7), viability of bacterial cells (colony-forming units per unit mass of film, Figure S8), and protein expression levels (Figures S10) were all measured. In each case, we found similar results for the E6-AT and CE6-AT films.

**Figure 3:**
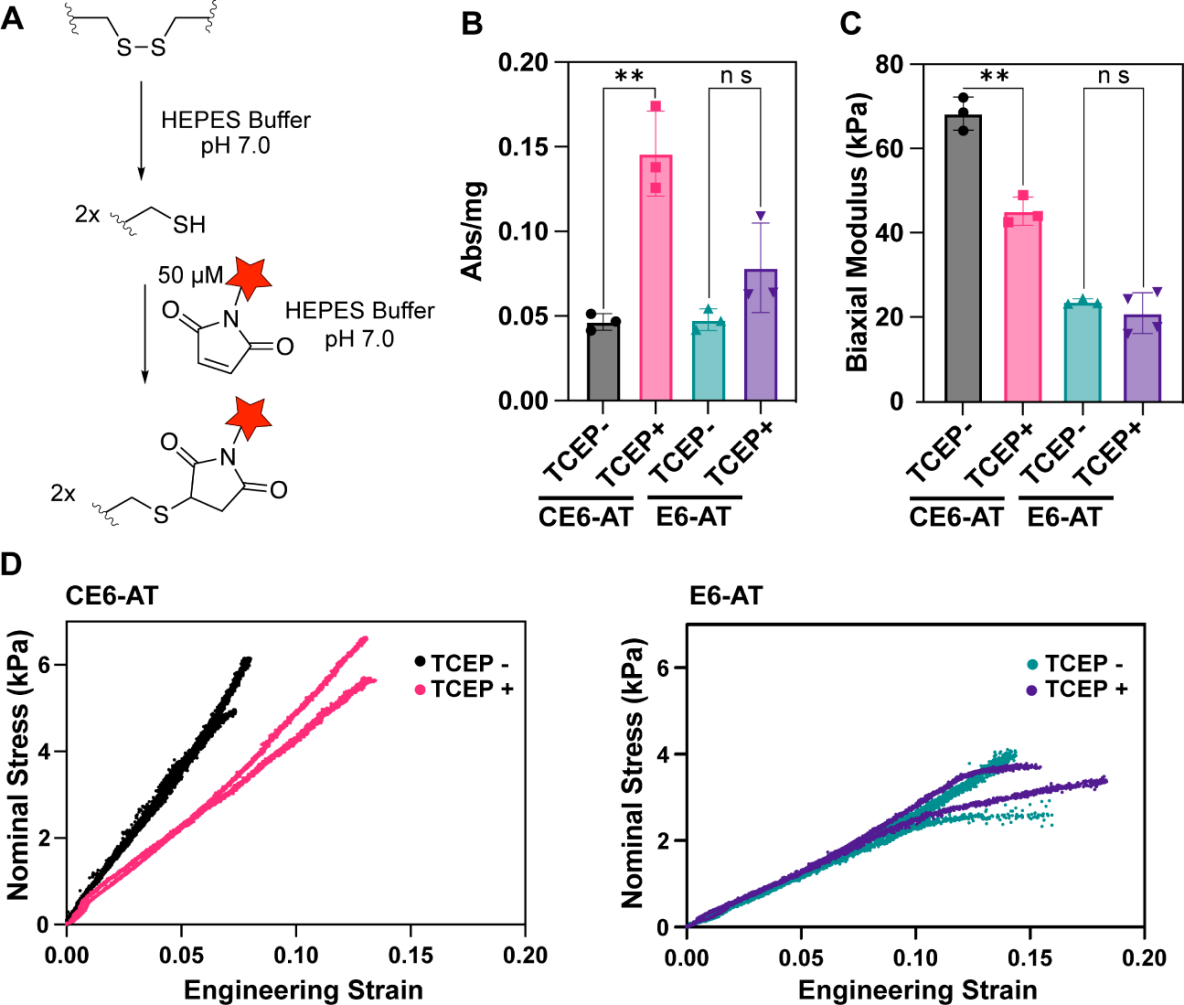
Disulfide bonds play an essential role in enhancing the mechanical properties of CE6-AT films. (A) Schematic of TCEP reduction of disulfide bonds and dye-labeling of free thiols. (B) Absorption intensity after dye-labeling of CE6-AT and E6-AT films with or without treatment with TCEP. Number of replicates for each group: 3. Error bars represent one standard deviation. (C) Biaxial modulus values for E6-AT and CE6-AT films with or without treatment with TCEP. Number of replicates for E6-AT TCEP+ = 4; for all other groups *n* = 3. Error bars represent one standard deviation. (D) Stress versus strain curves of E6-AT and CE6 AT films with or without treatment with TCEP. Number of curves shown for each group: 2. ** represents statistically significant differences in mean values with *p* < 0.01. ns indicates that differences in mean values are not statistically significant.

Disulfide bonds can be cleaved with a variety of reducing reagents. We chose odorless tris(2-carboxyethyl)phosphine (TCEP) to reduce and count disulfide bonds in bacterial films. Free thiols before and after reduction can be capped with a maleimide dye (Figure 3A). If CE6-AT forms disulfide bonds, then CE6-AT films treated with TCEP should exhibit higher maleimide-dye labeling than either CE6-AT without TCEP treatment or E6-AT films irrespective of TCEP treatment. We used 50 mM TCEP in 20 mM HEPES pH 7.0 buffer to treat preweighed films for 1 h (TCEP+ samples). Control films were not treated with TCEP but were immersed in 20 mM HEPES buffer at pH 7.0 for 1 h (TCEP- samples). The respective buffers were then removed, and films were treated with 50 μM Dylight 633-maleimide in HEPES buffer at pH 7.0 for 30 min. Films were rinsed three times with 500 μL of HEPES buffer and then lysed. The absorbance of the lysate at 633 nm was measured and normalized by the wet mass of the treated film. CE6-AT films treated with TCEP exhibited the strongest labeling (Figure 3B), suggesting that these films contain the largest concentration of free thiols (while CE6-AT and E6-AT films had similar Dylight-maleimide absorbance in the absence of TCEP treatment). This result suggests that surface-displayed thiols in CE6-AT films are predominantly in the oxidized (disulfide) form. We note that this procedure reveals total disulfide bonds; the percentage of intercellular disulfide bonds cannot be determined by this method.

We estimated the number of CE6-AT proteins per cell by assuming that the difference in labeling intensity between the CE6 TCEP+ group and the E6 TCEP+ group represents reduced CE6-AT proteins. The calculation method, discussed in Supporting Note 4 and Figure S9, yields an estimate of 2.5 × 10^5^ CE6-AT proteins per cell. This result agrees with those of quantitative Western blotting (Supporting Note 5 and Figure S11), which also gives values of around 2.5 × 10^5^ E6-AT and CE6-AT proteins per cell. Assuming the surface area of a single *E. coli* cell is 6 μm^2^^36^ the density of surface-displayed engineered protein is approximately 4 × 10^4^ proteins per μm^2^. Ajo-Franklin and coworkers recently reported a *C. crescentus*-based living material^25^ with RsaA fused to an elastin-like-peptide displayed at a density of 1.4 × 10^5^ proteins per μm^2^, which yielded concentrated cell suspensions with shear storage moduli of ∼14 kPa.^37−39^ The display density estimated here for E6-AT and CE6-AT films is consistent with these results, as is the modulus we’ve measured for CE6-AT. For a given material, the biaxial modulus is greater than Young’s modulus, which is typically three times the shear modulus, making the 44 kPa biaxial modulus of CE6-AT comparable to the 14 kPa shear modulus reported by Ajo-Franklin and coworkers.

Next, we addressed the question of whether disulfide bonds are responsible for the enhanced mechanical properties of the CE6-AT films. We treated CE6-AT and E6-AT films for 30 min either with 50 mM TCEP in 20 mM HEPES buffer at pH 7.0 (TCEP+) or with 20 mM HEPES buffer at pH 7.0 (TCEP- control). Ramp bulge tests were then performed on both the TCEP+ and TCEP- groups in 20 mM HEPES buffer at pH 7.0. TCEP appeared to have a minimal effect on the mechanical properties of E6-AT films, while CE6-AT films were very sensitive to TCEP treatment. As shown in Figure 3C, after the treatment of CE6-AT films with 50 mM TCEP for 30 min (a 1 h treatment was attempted, but it made CE6-AT too fragile to load into the bulge test apparatus), the biaxial modulus dropped from above 70 kPa to just slightly above 40 kPa. Interestingly, even after TCEP treatment, CE6-AT films still showed no evidence of yielding or failure under the conditions used for bulge testing (Figure 3D) and exhibited biaxial moduli larger than those of E6-AT films (Figure 3C). These results suggest that the TCEP reduction is incomplete after 30 min. OCT imaging revealed no structural changes in reduced films.

### Viscoelastic Behavior of Bacterial Films

**Figure 4:**
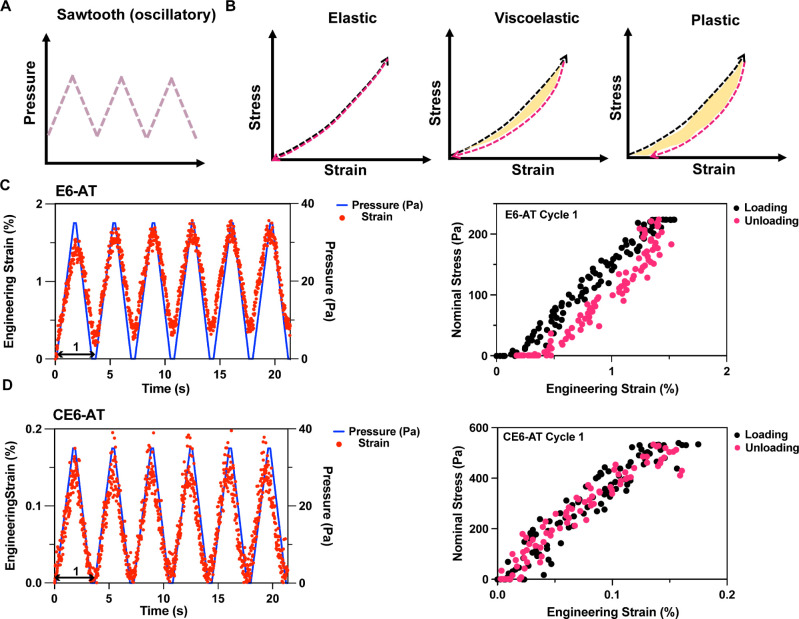
Viscoelastic behavior of bacterial films revealed by oscillatory bulge test. (A) Schematic diagrams of a ramp-up/ramp-down pressure cycle. (B) Response of elastic, viscoelastic, and plastic materials upon loading (black) and unloading (magenta). The area enclosed (yellow highlight) reveals energy lost per loading–unloading cycle. (C) Mechanical behavior of the E6-AT film in the oscillatory bulge test. Left: strain versus time curve superimposed with pressure versus time curve. Cycle 1 of loading is labeled by a double-headed arrow. Right:loading and unloading stress versus strain curves in the first cycle. (D) Mechanical behavior of the CE6-AT film in the oscillatory bulge test. Left: strain versus time curve superimposed with pressure versus time curve. Cycle 1 of loading is labeled by a double-headed arrow. Right: loading and unloading stress versus strain curves in the first cycle. (Note that the stress–strain curves in part D would appear nearly vertical on the scale used in part C due to the range of stress in (D) being 2.4 times that in (C), and the strain range in D being 1/10 that in (C)).

We investigated the elasticity and changes in properties over multiple loading cycles using an “oscillatory” bulge test. Using the syringe pump, we applied an approximately “sawtooth” loading/unloading pattern (Figure 4A–D, left, blue; see the Materials and Methods section for further details) and observed the biofilm response using OCT. The bulge test, data acquisition, and image analysis proceeded as previously described for the ramp test.

Elasticity is the ability of a material to return to its original size and shape upon the removal of applied loads.^40^ A perfectly elastic material returns to its original size and shape after a loading–unloading cycle with either a linear or nonlinear stress–strain response that is independent of the loading rate (Figure 4B, left: stress–strain curves for loading and unloading superimpose with no phase lag between stress and strain). A viscoelastic material, on the other hand, has characteristics of both solid and liquid, with properties that depend on the loading rate (Figure 4B, middle: the stress–strain response follows different paths for loading and unloading, and the area between the curves signifies energy dissipated each cycle). Within limits, viscoelastic materials can retain their properties cycle after cycle;^41^ in contrast, viscoplastic materials accumulate permanent deformation^42^ (Figure 4B, right; nonzero *x*-intercept after unloading).

CE6 films demonstrated an elastic response over multiple loading cycles (first cycle in Figure 4D; additional cycles in Figure S11). Under identical loading conditions (which for E6 resulted in a stress roughly half that for CE6 and a strain response that was 10 times that of CE6), E6 biofilms demonstrated initial plastic deformation in the first cycle followed by near-elastic behavior in the following cycles. E6 also showed a decreasing hysteresis with progressive loading cycles. Due to the soft nature of E6, conditions could not be identified that fell within the linear elastic regime and did not result in permanent deformation. The E6-AT film clearly shows viscoplastic behavior, failing to return to zero strain when the stress goes to zero (Figure 4C, left: the minimum strain progressively increases). Cycle 1 shows the characteristic nonzero strain after unloading (Figure 4C, right), and subsequent cycles begin from a nonzero strain and end at even greater strain (Figure S11). In contrast, a CE6-AT film returns to a zero strain cycle after cycle; there is no sign of yielding (Figure 4D, left).

### Self-Healing of CE6-AT Films

**Figure 5:**
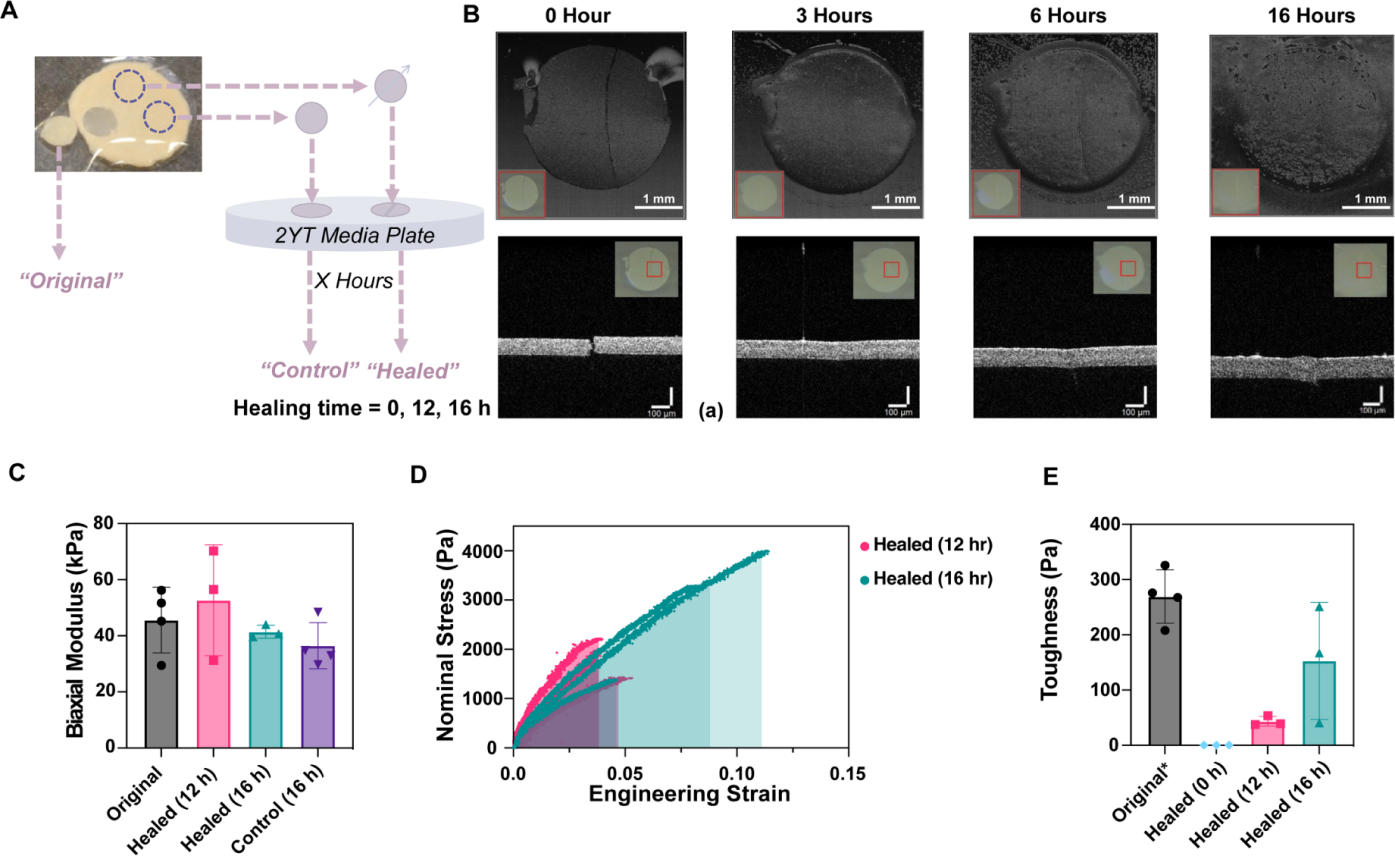
Self-healing of CE6-AT films. (A) Schematic of the healing experiment. (B) OCT scans of CE6-AT films healing on a 2YT plate show rapid healing of the injured bacterial film. The top row is exported as 3D renders. Scale: top, 4 × 4 mm scan box; bottom, 1 × 1 × 1 mm scan box. Insets are OCT camera images, manually cropped to the region being scanned. The red rectangular outline represents the scan box. (C) Biaxial modulus values of original, 12 h-healed , 16 h-healed, and control films. The number of replicates for each group: 4, 3, 3, 4, respectively. Error bars represent one standard deviation. None of the differences in modulus are statistically significant. (D) Stress versus strain curves of 12 h healed CE6-AT films and 16 h healed CE6-AT films. The number of curves shown for both time points is 3. (E) Toughness values of 0, 12, and 16 h healed films. For “Original*”, the asterisk indicates that these values are a lower bound on toughness as original CE6-AT films did not fail in the range of stresses tested. Number of replicates for 0, 12, and 16 h healed films is 3; for “Original*”, 4. Error bars represent one standard deviation. The difference in average toughness for the 12 and 16 h samples is not statistically significant.

An attractive feature of living materials is their potential to repair themselves. The ease of manipulating CE6-AT, with its elastic character and resistance to plastic deformation, enabled tests of its potential to heal after damage. 3-mm punches from CE6-AT films were separated from their polycarbonate filters and directly placed on fresh 2YT agar plates, which provide more nutrition than LB plates and were expected to promote enhanced bacterial growth. A 25-μm thick TEM grid was used to make a 3 mm cut through each test specimen (control films were placed on fresh 2YT agar plates without injury for comparison). Films were allowed to grow on the new plate at 37 °C and monitored to assess the healing process. OCT images of the cut films and the control films were acquired at 3, 6, and 16 h (Figure 5A) using a sample holder that enabled the Petri dish to be placed at the same position and orientation (Figure S12) to image approximately the same region each time. As negligible changes occurred over a time of several seconds, full 3D OCT images could be acquired. In OCT images of cut specimens, the initial injury was readily visible, both in 3D renderings of the exposed surface (Figure 5B, top, left) and in cross-sectional views (Figure 5B, bottom, left). OCT images of a control film are presented in Figure S13. The OCT images showed evidence of healing in as little as 3 h (Figure 5B); however, the time scale of recovery of mechanical properties could only be assessed by the bulge test.

We attempted to perform bulge tests at 6, 12, 16, and 24 h time points. Properties of the immediately cut biofilm are not reported, since cut films could not sustain any applied pressure difference (Figure S15B); fluid simply flowed through the cut to the other side. This feature was also used in our experiments to determine the moment when the film failed, also visible by OCT. For healing times of 6 h and longer, statistics are reported in Table S3. We discovered that healed biofilms could be separated from the nutrient plate by gently flooding the plate with PBS, and then allowing 1–2 min for the specimens to separate spontaneously from the 2YT agar underneath. Despite the gentle nature of this step, a few samples failed at the cut site (second column, Table S3). Of the intact samples, a few samples failed during the initial pressure equilibration step (third column, Table S3). The final column in Table S3 details whether films failed during the bulge test itself.

In comparing the various healing times, we discovered that films healed for either 6 or 24 h could be released from the agar and placed in the bulge-testing device; however, the small transient differences in pressure while filling the device were sufficient to break the film once again. Films healed for 12 and 16 h could be readily tested and exhibited biaxial moduli that were similar to original (CE6-AT, Figure 2E) and uninjured controls (Figure 5C). The moduli after 12 and 16 h healing were not significantly different (12 h: 52.7 ± 11.4 kPa; 16 h: 41.4 ± 2.31 kPa). In contrast to uninjured CE6-AT films, which survived the entire range of stresses tested (Figures 2 and 3), healed CE6-AT films failed at stresses ≤ 4 kPa (Figure 5D and Supporting Note 6).

Having accessible failure stresses for 12–16 h healing times enables us to evaluate toughness, the integral under the stress–strain curve (Figure 5D). Despite substantial variability in the 16 h samples, there was a clear increase in toughness from 12 to 16 h healing time (Figure 5E). Because the original films did not fail at the stresses we accessed in our bulge-test apparatus, we do not know their toughness. However, we can calculate a lower bound on the toughness of CE6-AT films by measuring the area under stress–strain curves for the original samples. This calculation yields a value of 269 ± 48 Pa—much greater than the toughness of the healed samples (Figure 5E, 43 ± 9 Pa for 12 h and 153 ± 105 Pa for 16 h healed samples).

We observed that failure occurred at the location of the original defect. This phenomenon made us wonder if the defect area was characterized by a lower cell density than the undamaged parts of the film. To test this hypothesis, we grew films of CE6-AT cells with plasmids encoding mWasabi and performed fluorescence microscopy from 0 to 24 h after defect generation. We found that the original defect remains visible after 16 h of healing (Figure S15), consistent with the hypothesis that cell density in this region of the film has not fully recovered. We also measured the mechanical properties of CE6-AT films expressing mWasabi and found them to be indistinguishable from CE6-AT films without fluorescent protein expression (Figure S16). Further study will be needed to determine the roles of nutrient supply, cell motility, and cell proliferation in the recovery of film structure and properties.

Similar self-healing experiments were attempted for E6-AT films; however, even E6-AT films that appeared to be healed according to the OCT images (Figure S17) were too fragile for bulge-testing.

## Materials and Methods

### Bacterial Strains

All experiments on bacterial films were conducted with *E. coli* strain DH10B (Invitrogen, Carlsbad, CA). Purified E6-AT protein was expressed in *E. coli* strain BL21 (NEB, Beverly, MA). Details of reagents, cloning, and protein expression experiments can be found in Table S1 and Supporting Notes 1 and 2.

### Preparation of Bacterial Films

Individual colonies harvested from LB plates were grown overnight to the stationary phase in the liquid LB medium supplemented with 100 mg/L ampicillin to maintain plasmid stability. The resulting cultures were then diluted to an optical density at 600 nm (OD_600_) of approximately 0.8. Diluted cultures were loaded on UV-sterilized track-etched polycarbonate filters (Nuclepore Whatman, 0.2 μm pore size) mounted in vacuum filter units (Nalgene Rapid-Flow Thermo-Fisher). For 2.5 cm diameter polycarbonate filters, 200 μL culture volumes were loaded and vacuum filtered. After the filtration was complete, filters were transferred to fresh LB plates. Bacterial films were grown in a 37 °C incubator. Filters were moved to fresh LB agar plates containing suitable antibiotics every day for 7 days to allow for film maturation and mechanical testing.

### Erosion Assay

Seven-day old bacterial films grown on membrane filters were immersed in 7 mL of 1× PBS buffer at pH 7.4 in the wells of 6-well plates (Corning, Thermofisher). The 6-well plate was then placed on a rocking platform (Bio-Rad) set at a 15° tilt angle and 15 rpm frequency. The OD_600_ of PBS in contact with the bacterial film was measured at 1 and 24 h.

### Microtomy of Bacterial Films

Seven-day old bacterial films on polycarbonate filters were cut with a 6 mm diameter biopsy punch (Miltex). The circular section of the film was embedded in Tissue-Tek resin (Sakura) and frozen at −20 °C overnight. The frozen piece was microtomed at −20 °C with each section at 50 μm thickness. Microtomed sections were placed on glass slides for staining and imaging.

### Flow Cytometry

E6-AT films were scraped and transferred to an Eppendorf tube and blocked for 30 min with agitation (3% BSA in PBS). Cells were then centrifuged and resuspended in staining solution (5 μg/mL Anti-His conjugated Dylight 488 Antibody (HIS.H8 Thermofisher), and 1% BSA in PBS). This solution was then agitated for 1 h, after which the cells were washed three times in PBS. Cells were strained through a 40 μm filter to remove aggregates and run on a MoFlo XDP cell sorter (Beckman Coulter, Inc.) equipped with a 488 nm laser. Flow cytometry data were analyzed by using MATLAB.

### Microtome Staining

For antibody staining, microtome sections on glass coverslips were blocked with 3% BSA in PBS for 0.5 h under static conditions. The blocking buffers were then removed with a Kimwipe paper tissue. A staining solution (5 μg/mL Anti-His conjugated Dylight 488 Antibody (HIS.H8 Thermo Fisher), 1% BSA in PBS) was dropped on the coverslip, and the solution was incubated for 1 h in a dark chamber. The residual staining solution was removed with a Kimwipe, and microtome sections were washed three times with 1% BSA in PBS before imaging. The microtome sections on coverslips were then placed between coverslips and slides separated by a 120-μm spacer (SecureSeal, VWR).

### Bacterial Film Lysis

Bacterial films were scraped from the polycarbonate filter and lysed in 4% SDS 1× PBS pH 7.4 at 100 °C for 30 min on a thermo shaker (VWR Scientific) at 900 rpm in an Eppendorf tube. For 3–6 mg of bacterial film mass, 500 μL of lysis buffer was added.

### Quantification of Colony Forming Units

The bacterial film was scraped from the polycarbonate filter and weighed in an Eppendorf tube. A 500 μL volume of 1× PBS at pH 7.4 was added to the tube. The film was first agitated by pipetting up and down until it was broken into small pieces and vortexed at least three times until no large fragments (mm-sized) could be observed and the suspension became turbid. The suspension was then serially diluted 10 to 10^6^-fold in a 96-well plate. Three 10 μL drops of each dilution were dropped on an LB agar plate and incubated overnight. A plate with 10 to 100 colonies was selected, and the number of colonies on the plate was counted to allow calculation of the number of colony forming units (CFU) in the original bacterial film.

### Bulge Test Device Assembly

Clear acrylic sheets (1/16 in. thickness, McMaster-Carr) were used to fabricate the millifluidic device. Three rectangles (10 cm × 2.5 cm; dimensions chosen such that the device with its reservoirs would fit under the OCT objective) were cut by using a laser cutter (Industrial Laser ILS 9.75)). One served as the base of the device and was bonded with epoxy adhesive to another one in which the sample chamber and channel to connect to “Reservoir 2” had been laser cut/ablated (Figures 2A and S5), enabling control of the pressure below the sample when the device is assembled. The third acrylic rectangle had two circular holes, one to permit fluid from “Reservoir 2” to pass through and the other to allow fluid from “Reservoir 1” to enter a channel leading to the sample chamber, enabling the control of the pressure on the top face of the sample. The channel above the sample was sealed with a glass coverslip to enable imaging. The two resulting parts could be assembled reversibly, as described below. For the reservoir connections to the top face, thicker acrylic slabs with through holes threaded to accept quick-tube coupling fittings (51525K442, McMaster-Carr) were bonded to the top surface. The reservoirs themselves were syringes of a known internal diameter and total volume.

### Sample Loading Protocol for the Bulge Test

A polycarbonate filter with a bacterial film grown on top was placed in a Petri dish containing the sterile buffer used for bulge testing (either PBS or HEPES, demonstrated to give indistinguishable results). A 3 mm diameter biopsy punch (Integra Biosciences) was used to make a circular cut through the bacterial film but not through the polycarbonate. A sample support disk (copper TEM disk, 1.5 mm diameter circular aperture, 3.05 mm outer diameter, thickness 25 μm, Ted Pella) was then inserted between the bacterial film and the polycarbonate from the outer edge of the film and used to gently separate the film from the polycarbonate. Once the edge of the film was freed, the cut 3 mm bacterial film disk freely floated away from its polycarbonate support and could be lifted out of the buffer using the support disk and placed into the bottom chamber of the device (prefilled with buffer). A second support disk was placed on top to sandwich the bacterial film, followed by an O-ring (Precision Associates, Inc.). Prior to sealing the two halves of the device together, a 5 mL syringe was filled with buffer and capped with a 30G needle connected to thin silicone tubing (0.31 mm ID, 0.64 mm OD, HelixMark). The tubing was threaded through into the Luer socket on the top half of the device and placed along the top channel using forceps such that the tube terminated in the viewing window of the chamber that would eventually be directly above the sample. Following this, the two chamber halves were sealed by using a thin layer of vacuum grease. The two chambers were then filled simultaneously with buffer solution: the upper channel using the tubing connected to the syringe filled with buffer and the lower channel by way of the associated Luer slip connector, drop by drop, until both connectors were filled, following which the tubing was gently extracted and two reservoirs were attached to the Luer slip connectors on either side. Syringes used as reservoirs in the collected data sets were 60 mL syringes (BD Scientific) with ID = 26.72 mm. The apparatus was rinsed with soap and DI water and allowed to dry between uses; separate reservoirs and syringes were used for different buffers.

### Bulge Test Pressure Profile Control

A syringe pump was used to impose various pressure–time profiles in these experiments. “Ramp” and “sawtooth” profiles involved PBS addition at a flow rate of 70 mL/min, with a final target volume of 55 mL in one ramp bulge test and 2 mL in each cycle of an oscillatory bulge test unless otherwise specified. The flow rate was chosen to be the maximum accommodated by the syringe pump to minimize perfusion through the biofilm during the experiment. When performing cycles of infusion and withdrawal (typically 20–30 cycles), we noted that the profile is not precisely a sawtooth, as the syringe pump did not reverse direction instantaneously. A separate experiment was performed to characterize the actual infusion/withdrawal transient of the pump using a 240 fps video of the fluid level in a graduated cylinder. This yielded the following profile: infusion (2 mL over 1.576 ± 0.008 s), dwell at maximum (0.20 ± 0.01 s), withdrawal (2 mL over 1.431 ± 0.008 s), and dwell at minimum (0.34 ± 0.02 s).

As the film response to the pump transient was measured separately from the pump transient, a correction was applied to synchronize the start of the OCT imaging with the pressure profile. A full cycle of the syringe pump took 3.547 s, in accordance with the transient strain response of CE6, which was approximately elastic. The pump profile showed that each minimum lasted for 18 time points for Δt = 0.019 s. Thus, an initial delay of 18/2 × 0.019 s = 0.17 s was added prior to the start of the first infusion.

### OCT Imaging

All OCT imaging was performed with a Thorlabs OCT (GAN210 base unit: 930 nm central wavelength, 6/4.5 μm axial resolution in air/water, 2.9/2.2 mm imaging depth (air/water), OCTP-900 scan head, and OCT-LK3-BB scan lens: 36 mm FL, 8 μm lateral resolution). The A-scan/line rate was 36 kHz for all measurements (acquisition time = 19 ms). Biofilm thicknesses were calculated by assuming a refractive index of 1.4. This value was based on both the manufacturer’s recommendation and the literature on *E. coli*.^43^

### OCT Alignment for Self-Healing Experiments

To image comparable regions of each sample throughout the healing experiment, we 3D-printed a Petri dish holder (Figure S19) and secured it to the OCT base plate with a cavity that provided a snug fit for a single 60 mm diameter Petri dish. The holder was at roughly a 10° slant to avoid OCT image artifacts from reflections at the air–biofilm interface. One 3 mm diameter biofilm sample was placed at the center of each Petri dish, and permanent marks on both the Petri dish and holder enabled alignment of the Petri dishes each time they were removed from the incubator and placed in the OCT system for imaging. Although the vertical focus of the OCT needed to be adjusted each time, the scan settings in the XY plane were not changed to ensure that the scanned region was consistent for a given sample. Alignment was confirmed using the inspection camera images from the ThorImage OCT software, which provided an overall view of the sample as well as the position of a specific “scan box” (Figure 5B, camera image insets with red rectangular boundaries).

### OCT Image and Mechanical Property Analysis

During a pressure ramp experiment (Figures 2 and 3), the typical imaging process acquired over 2500 2D OCT images showing the evolution of a biofilm cross-section throughout the experiment. The features that could be captured were limited by (i) perfusion, which in most cases was negligible, (ii) the maximum fluid held by the reservoir, which was 70 mL, and (iii) the thickness of the samples tested. Thicker samples experience less stress at a given hydrostatic pressure, which means that the failure of a particular sample could not always be captured. A typical analysis protocol resulted in the stress–strain curve, as shown in Figure S18. The analysis protocol followed the assumptions of membrane theory, i.e., that (i) the thickness of the membrane is small in comparison to the in-plane dimensions, (ii) the bending stiffness is negligible, and (iii) due to (i) and (ii), in-plane stresses are assumed to be constant throughout the thickness of a membrane. Additionally, we assumed (i) that there is an idealized equibiaxial deformation throughout the entire inflated membrane. In practice, this is only true at the pole, as the deformation transitions to a constant width elongation at the clamped edge; also (ii) that stress could be estimated using a spherical cap assumption, and strain could be estimated by changes in arc length of the deformed biofilm.

Following the assumptions outlined above, the stress and strain states are as follows:
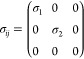
1
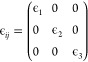
2

We assume here that σ_1_ = σ_2_ = σ and ϵ_1_ =ϵ_2_ = ϵ cross the entire membrane. Applying Hooke’s law, the relationship between σ and ϵ is
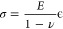
3

where  is the biaxial modulus of the material, sometimes referred to as the “modulus” in the text, and evaluated from the linear regions of each plot. We do not report the two quantities *E* and *v* independently since they cannot be deconvolved. We assume comparable Poisson’s ratio across all samples.

2D data sets were exported as tiff files and cropped using ImageJ. The resulting images were processed with in-house MATLAB scripts: the images were binarized; the top and bottom surfaces of the film were detected based on changes in pixel intensity and fit to fourth-degree polynomials, which were then used to estimate the arc lengths of the top and bottom surfaces of the film (Figure S18).

Engineering strain ϵ was calculated as the change in the arc length divided by the original arc length for both the top and bottom surfaces. The two values were averaged.

Nominal stress was calculated using the equation for stress in a thin-walled spherical pressure vessel:
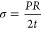
4where σ is film stress, *P* is the applied pressure difference (greater inside the sphere), *R* is bulge radius of curvature, and *t* is film thickness. Furthermore, the radius was approximated using the known radius *a* of the aperture and the height *h* of the center of the film, directly measurable in the OCT image, as . The ratio of the aperture diameter 2*a* to film thickness *t* ranged from 16.6 to 25. Although this ratio should be >20 when applying the membrane approximation, the fact that the top and bottom surfaces of the biofilm did not show significant differences in their stress–strain behavior (Figure S19) suggests that it is a reasonably good approximation in the present bacterial films. The film stress was evaluated using R for the top and bottom surfaces of the film, and the two values were averaged. True stress and strain use instantaneous thickness for calculation, while nominal stress and engineering strain use the initial thickness. True stress and strain were used for Figure 2C,D; nominal stress and engineering strain were used for other figures.

### Fluorescence Imaging

The fluorescence images of processed samples with fluorescent labels were obtained with a 63X, 1.518 N.A. oil-immersion objective on a Zeiss LSM 880 confocal microscope (Carl Zeiss AG, Oberkochen, Germany) at the Caltech Biological Imaging Facility. Single-photon confocal laser scanning imaging was performed with 488 and 591 nm lasers in the mWasabi (λex/λem: 493/509 nm), mCherry (λex/λem: 587/610 nm), and Dylight 488 channels (λex/λem: 493/518 nm). The images were visualized and analyzed with a Fiji or Imaris Viewer (Oxford Instruments, Abingdon, UK).

### Image Processing and Data Analysis

Image color-coding of confocal fluorescence microscopic images was done using ImageJ. Intensity normalization of the fluorescence image z-stacks was also done in ImageJ. 3D rendering of z-stacks was performed in either ImageJ or Zeiss Zen Blue. Data plotting and analysis of the OCT images were performed in MATLAB (MathWorks) and Prism (GraphPad).

### Statistics and Reproducibility

For most experiments creating the micrographs reported herein, the data generated were in triplicate. The numbers of replicates for experiments are specified in the figures and Supporting Information.

## Conclusions

Bacteria outfitted with surface-displayed associative proteins can assemble and grow into matrix-free cohesive films on perforated solid supports. When unstructured elastin-like peptides were displayed at the cell surface, the bacterial films were soft and viscoplastic with biaxial moduli of approximately 14 kPa. These films yield easily and fail when film stress reaches 1 to 2 kPa. The addition of a cysteine residue to the N-terminal region of the elastin-like-peptide causes the biaxial modulus to increase 3-fold and eliminates yielding and failure under our testing conditions. We reduced the cysteine-containing films chemically with TCEP and observed reduced mechanical strength, supporting the hypothesis that covalent disulfide cross-links contribute to the enhanced mechanical properties. These results demonstrate that the mechanical properties of films made by protein-mediated bacterial assembly can be manipulated genetically and that single-site mutations in protein sequence can markedly change the mechanical properties. These living bacterial films self-heal within 16 h after injury. The living material system introduced here is characterized by autonomous assembly, genetically encoded mechanical properties, and recovery after damage. We believe that such materials may find application in biocatalysis, environmental sensing, and bioremediation. Much remains to be learned about the long-term behavior of bacterial films and about strategies for film stabilization, including protection from desiccation and the engineering of films capable of controlled sporulation.

## Supporting Information Available

Detailed description of Videos S1–S5 (PDF)
Additional experimental information including tables of plasmid constructs and protein sequences, additional microscopy images, Western blotting and mass spectrometry data, and bulge test device setup and image analysis details (PDF)
Videos of OCT-scanned “Ramp” bulge tests of E6-AT and CE6-AT films, peeling experiments, and the only observed failure event of a CE6-AT film (ZIP)
