# Supplementary material for: Cohesive Living Bacterial Films with Tunable Mechanical Properties from Cell Surface Protein Display
Source: ACS Synth Biol. 2024 Nov 1;13(11):3686–97. doi: 10.1021/acssynbio.4c00528 (PMC11574920; doi:10.1021/acssynbio.4c00528)
Supplement: Supplementary file 1 — sb4c00528_si_002.pdf [file sb4c00528_si_002.pdf]

Video Caption

Supplementary Video 1: Ramp Bulge Test of E6-AT Film. Bulge testing of E6-AT film with pressure ramp rate of 20.4 Pa/s, imaged by OCT. Video plays at actual rate of bulge test.

Supplementary Video 2: Ramp Bulge Test of CE6-AT Film. Bulge testing of CE6-AT film with pressure ramp rate of 20.4 Pa/s, imaged by OCT. Video plays at actual rate of bulge test.

Supplementary Video 3: Peeling of E6-AT Film from the Polycarbonate Filter. E6-AT films are soft and difficult to remove from the filter.

Supplementary Video 4: Peeling of CE6-AT Film from the Polycarbonate Filter. CE6-AT films are cohesive and easy to remove.

Supplementary Video 5: Failure of CE6-AT Film. Failure of a CE6-AT film which was left in PBS buffer for 4 h. A ramp bulge test with pressure ramp rate of 20.4 Pa/s was applied to this film. Video plays at a rate 10 times lower than the actual testing rate to show the failure process more clearly.
