# Supplementary material for: Cohesive Living Bacterial Films with Tunable Mechanical Properties from Cell Surface Protein Display
Source: ACS Synth Biol. 2024 Nov 1;13(11):3686–97. doi: 10.1021/acssynbio.4c00528 (PMC11574920; doi:10.1021/acssynbio.4c00528)
Supplement: Supplementary file 2 — sb4c00528_si_003.pdf [file sb4c00528_si_003.pdf]

Supporting Information for

Cohesive Living Bacterial Films with Tunable Mechanical Properties from Cell Surface Protein Display

Hanwei Liu †, Priya K. Chittur †, Julia A. Kornfield, David A. Tirrell*

Division of Chemistry and Chemical Engineering, California Institute of Technology, Pasadena, California 91125, United States

† These authors contributed equally: Hanwei Liu, Priya Chittur

*Corresponding author. Email: tirrell@caltech.edu

This PDF file includes:
Figs. S1 to S19
Tables S1 to S3
Notes S1 to S6
References

Supplementary Note 1: Reagents and suppliers

Restriction enzymes, ligase, and Q5 DNA polymerase were purchased from New England Biolabs (Beverly, MA). DNA oligos and G-blocks were purchased from Integrated DNA Technologies (Coralville, IA).

Supplementary Note 2: Plasmid subcloning

Recombinant fusion proteins were produced by standard recombinant DNA technology. *E. coli* strain DH10B was used for all cloning steps and material preparation.

Genes encoding the autotransporter protein along with elastin solubility/stability tags have been previously cloned by our group into modified pQE-80L plasmids.[1] All plasmids used in this work were derived from the pAT-ST plasmid[1], which encodes a SpyTag peptide at the N-terminus fused with the transmembrane domain of EhaA at the C-terminus. In such constructs, the N-terminal peptide will be displayed at the cell surface and the C-terminal domain is inserted into the outer membrane of *E coli*.

Plasmid pX-E6[1], which encodes a 150-amino acid elastin-like-protein, was digested with BamHI and XhoI and inserted into a similarly digested pAT-ST vector to yield pAT-E6. The T5 promoter of pAT-E6 drives constitutive expression of protein E6-AT.

pAT-ST[1] was mutated to encode a cysteine residue after the 6xHis tag and before the SpyTag peptide. The resulting plasmid was designated pAT-Cys-ST. pX-E6, encoding six 25-residue elastin-like repeats flanked by 5’ BamHI and 3’ XhoI sites, was digested with the corresponding enzymes and inserted into a pAT-Cys-ST plasmid digested with 5’ BamHI and 3’ XhoI to yield pAT-CE6. The T5 promoter of the plasmid drives constitutive expression of protein CE6-AT. Sequences of all constructs were confirmed by Laragen Inc (Culver City, CA).

Supplementary Note 3: Buffer recipe

The HEPES buffer used in this work contains 20 mM HEPES, 115 mM NaCl, and 1.2 mM $MgCl_2$ buffered at pH 7.0[2].

The PBS buffer used in this work was purchased from ThermoFisher Scientific and contains 155 mM NaCl, 1 mM KH_2PO_4 and 3 mM Na_2HPO_4, buffered at pH 7.4.

Table S1: Plasmids used in this study

Name	Backbone/origin/promoter	Purpose
pQE-Empty	pQE80l/colE1/T5	Empty plasmid for cloning and maintaining ampicillin resistance
pAT-E6	pQE80l/colE1/T5	Constitutive expression of E6-AT protein on cell surface
pAT-CE6	pQE80l/colE1/T5	Constitutive expression of CE6-AT protein on cell surface
pX-E6[1]	pQE80l/colE1/T5	Cloning of pAT-E6 and pAT-CE6
pAT-ST[1]	pQE80l/colE1/T5	Cloning of pAT-E6 and pAT-CE6
pAT-Cys-ST	pQE80l/colE1/T5	Cloning of pAT-CE6
pKPY680[1]	pBAD33/p15a/pJ23100	Constitutive expression of mWasabi
pKPY681[1]	pBAD33/p15a/pJ23100	Constitutive expression of mCherry

Table S2: Protein sequences

**Protein:**	Sequence (N-terminal amino acid first)
E6-AT	MKYLLPTAAAGLLLLAAQPAMAMRGSHHHHHHGSVDVPGA<br>GVPGAGVPGEGVPGAGVPGAGVPGAGVPGAGVPGEGVPGAG<br>VPGAGVPGAGVPGAGVPGEGVPGAGVPGAGLDVPGAGVPGA<br>GVPGEGVPGAGVPGAGVPGAGVPGAGVPGEGVPGAGVPGAG<br>VPGAGVPGAGVPGEGVPGAGVPGAGLETPTPGPDLNVDNDLR<br>PEAGSYIANLAAANTMFTTRLHERLGNTYYTDMVTGEQKQTT<br>MWMRHEGGHNKWRDGSGQLKTQSNRYVLQLGGDVAQWSQ<br>NGSDRWHVGVMAGYGNSDSKTISSRTGYRAKASVNGYSTGL<br>YATWYADDESRNGAYLDSWAQYSWFDNTVKGDDLQSESYK<br>SKGFTASLEAGYKHKLAEFNGSQGTRNEWYVQPQAQVTWMG<br>VKADKHRESNGTLVHSNGDGNVQTRLGVKTWLKSHHKMDD<br>GKSREFQPFVEVNWLHNSKDFSTSMDGVSVTQDGARNIAEIKT<br>GVEGQLNANLNVWGNVGVQVADRGYNDTSAMVGIKWQF
CE6-AT	MKYLLPTAAAGLLLLAAQPAMAMRGSHHHHHHCGSVDVPG<br>AGVPGAGVPGEGVPGAGVPGAGVPGAGVPGAGVPGEGVPGA<br>GVPGAGVPGAGVPGAGVPGEGVPGAGVPGAGLDVPGAGVPG<br>AGVPGEGVPGAGVPGAGVPGAGVPGAGVPGEGVPGAGVPGA<br>GVPGAGVPGAGVPGEGVPGAGVPGAGLETPTPGPDLNVDNDL<br>RPEAGSYIANLAAANTMFTTRLHERLGNTYYTDMVTGEQKQT<br>TMWMRHEGGHNKWRDGSGQLKTQSNRYVLQLGGDVAQWS<br>QNGSDRWHVGVMAGYGNSDSKTISSRTGYRAKASVNGYSTG<br>LYATWYADDESRNGAYLDSWAQYSWFDNTVKGDDLQSESY<br>KSKGFTASLEAGYKHKLAEFNGSQGTRNEWYVQPQAQVTWM<br>GVKADKHRESNGTLVHSNGDGNVQTRLGVKTWLKSHHKMD<br>DGKSREFQPFVEVNWLHNSKDFSTSMDGVSVTQDGARNIAEI<br>KTGVEGQLNANLNVWGNVGVQVADRGYNDTSAMVGIKWQF

Highlight Color Reference (starting from N-terminus):

Red: PelB leader peptide
Yellow: 6x His tag
Magenta: Cysteine
Blue: E6
Green: EhaA autotransporter

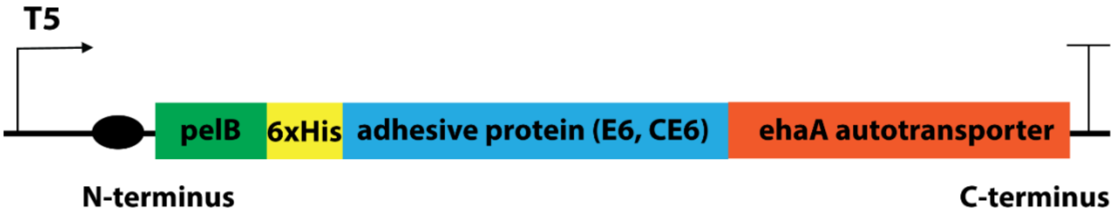

Figure S1. Expression construct for surface displayed adhesive proteins.

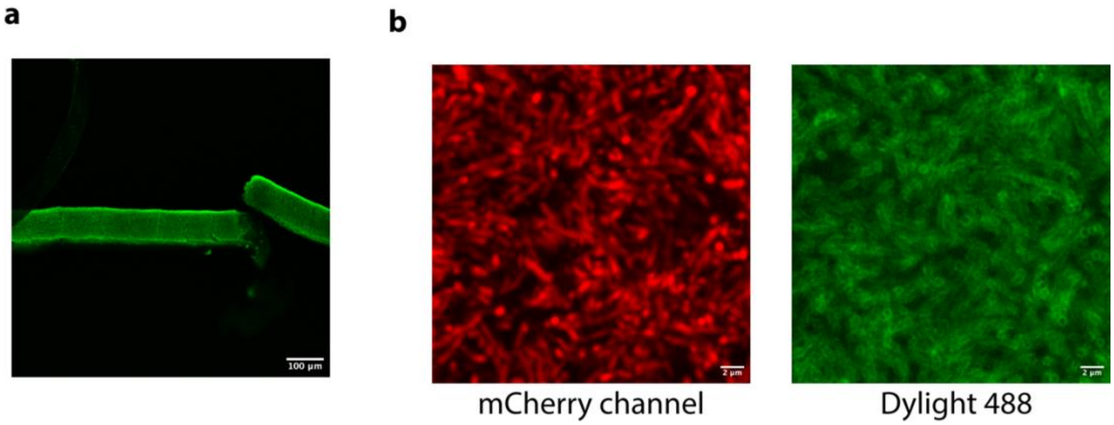

Figure S2. Antibody staining of E6-AT films. **a**, Microtome sections of E6-AT films were stained with anti-His tag antibody conjugated with Dylight 488. Expression of E6-AT protein across the full thickness is apparent. Scale bar, 100 μm. **b**, An E6-AT film was engineered to express mCherry and stained with anti-His tag antibody conjugated with Dylight 488. The mCherry channel shows cell packing in the bacterial film; the Dylight 488 channel shows expression of E6-AT at the cell surface. Scale bar, 2 μm.

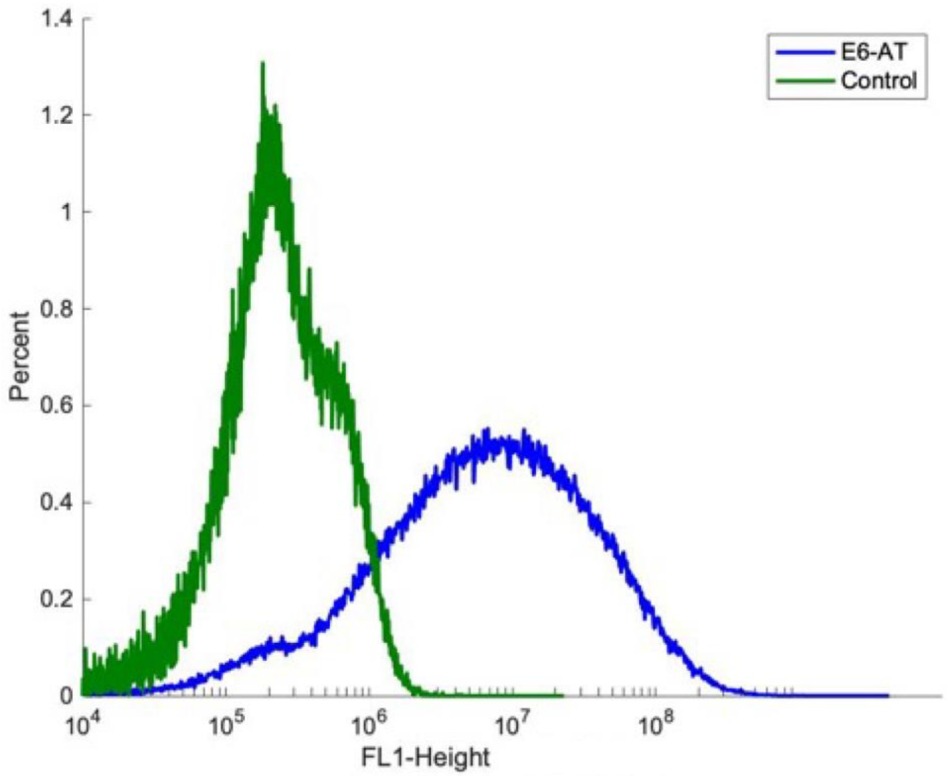

Figure S3. Flow cytometry of cells derived from E6-AT films. Flow cytometry enables relative quantification of protein expression. Control and E6-AT films were sampled and stained with anti-His tag antibody conjugated to Dylight 488 and analyzed by flow cytometry. X-axis values correspond to antibody labeling intensity; Y-axis values correspond to percentage of population. E6-AT exhibits stronger labeling than the control. CE6-AT could not be analyzed by flow cytometry because cells could not be adequately dispersed.

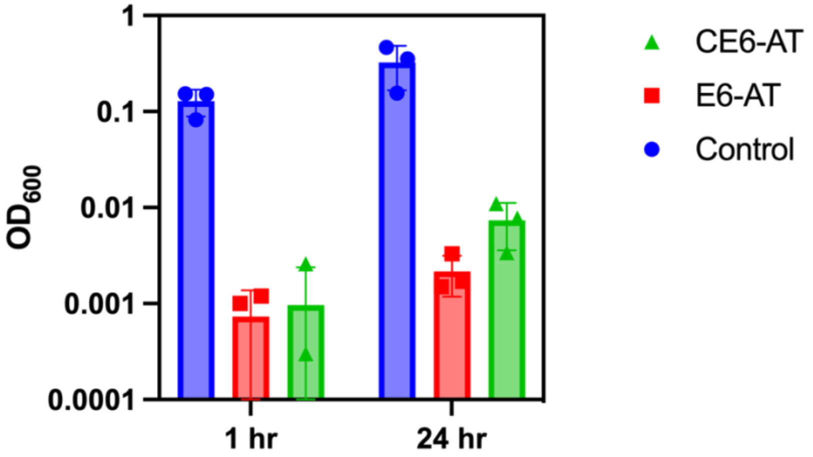

Figure S4. Erosion assay results for control, CE6-AT and E6-AT films. OD_{600} of PBS buffer was measured after 1 h and 24 h of rocking (15° angle, 15 cycles per minute).

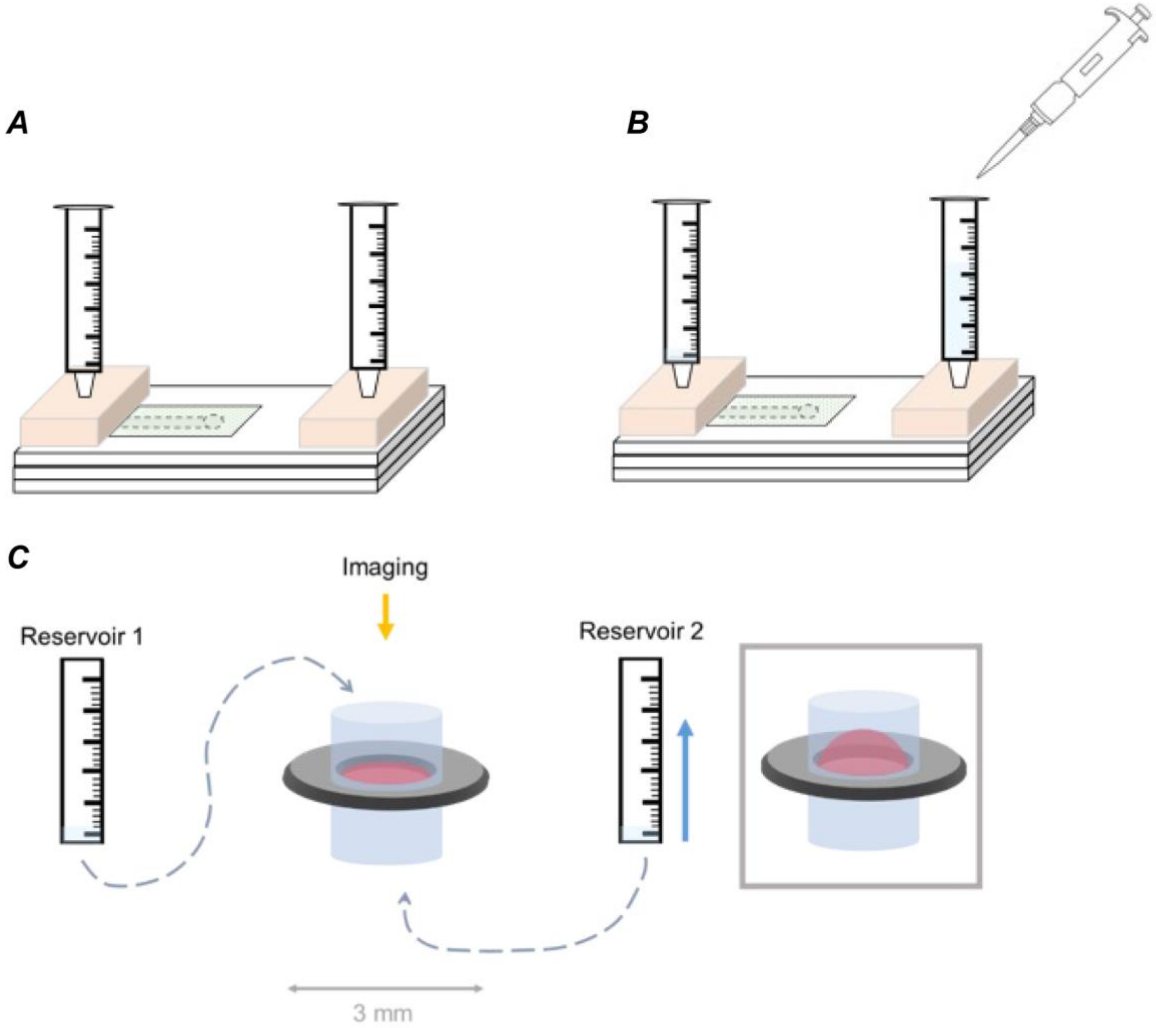

Figure S5. Schematic of reservoirs used to drive the bulge test. (A) The ports to the reservoirs shown in Figure 2A are connected to syringes that serve as fluid reservoirs. (B) As fluid is added to Reservoir 2, the hydrostatic pressure below the bacterial film increases. (C) The pressure difference across the bacterial film causes it to bulge upward through the central aperture (illustration in gray rectangle, right). In cyclic loading experiments, the pressure difference is reduced by adding fluid to Reservoir 1 until the fluid levels are matched, then repeating the cycle by adding fluid to Reservoir 2. In the present experiments, 60-mL syringes were used (5.61 cm^2 internal cross section), such that adding 858 μL of PBS increases the reservoir level by 1.53 mm, which increases the hydrostatic pressure on the corresponding face of the sample by 15.0 Pa.

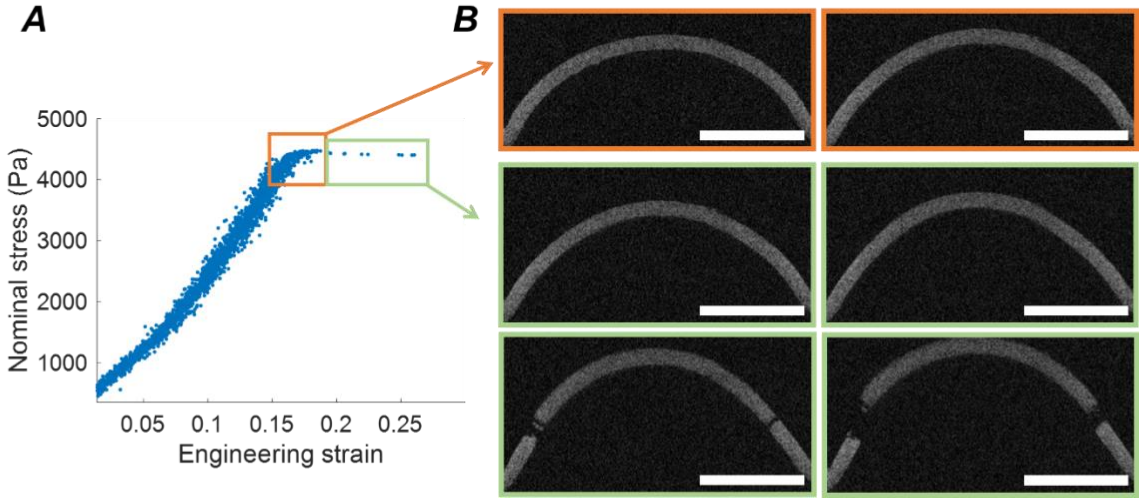

Figure S6. The only CE6-AT film that exhibited failure. This CE6-AT film was accidentally soaked in PBS for 4 h prior to testing. During testing, when the nominal stress reached approximately 4500 Pa, failure occurred abruptly. Regions of (A) the stress-strain curve correspond to physical features observable in (B) OCT cross-section images of the biofilm: the short region of decreasing slope of stress vs strain from 0.15 to 0.18 (A, orange rectangle) corresponds to a transition in film shape from a spherical cap to having stronger curvature near the apex (B, top), followed by failure within seven frames acquired at 16 ms intervals (A, green rectangle, elapsed time less than 0.2 s; representative images are in the middle row of B). The bottom row of OCT images were acquired after rupture, when strain could no longer be measured and the pressure difference was lost as fluid from Reservoir 2 flowed through the gaps in the film). Scale bar 500 µm. Unlike E6-AT films, which yield initially and then fail, the one CE6-AT film that failed exhibited brittle failure: a sharp crack rapidly propagated around the bulge.

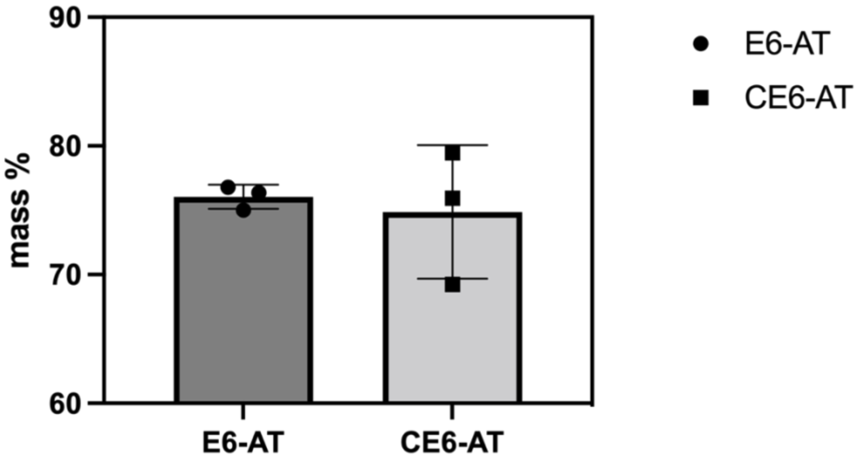

Figure S7. Water content of E6-AT and CE6-AT films. Both films contain roughly 75% water by mass, similar to the water content of *E. coli* cells.[3] Number of replicates: 3.

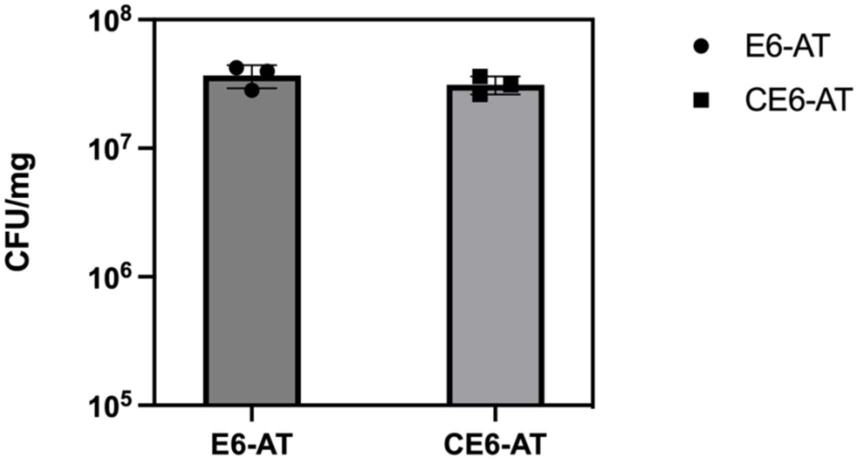

Figure S8. Colony forming units (CFU) per unit mass of E6-AT and CE6-AT films. Both films contain approximately $3*10^7$ CFU per mg of film. Number of replicates: 3.

Supplementary Note 4: Calculation of CE6-AT copy-number per cell using TCEP reduction

Using Beer's law,[4] we measured the absorbance versus concentration for Dylight 633-maleimide at 633 nm to prepare a calibration curve.

$$A = \varepsilon l c$$

A is absorbance; ε is molar extinction coefficient of the molecule; l is light path length; c is concentration of the molecule.

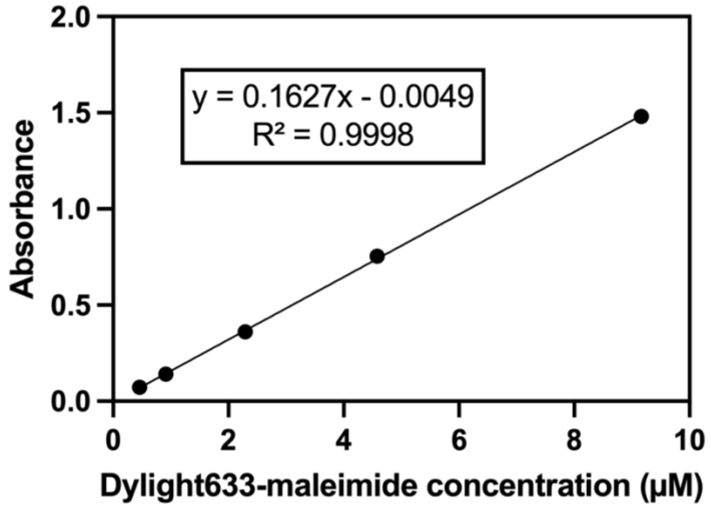

Figure S9. Calibration curve for Dylight 633-maleimide at 633 nm.

The calibration curve yields a molar extinction coefficient for Dylight 633 maleimide of 0.1627 $\mu M^{-1} cm^{-1}$. We assume that the difference in labeling intensity Δ between CE6-AT TCEP + and E6-AT TCEP + films is a result of reduction and labeling of CE6-AT by Dylight 633 maleimide. The calibration curve allows determination of the concentration of CE6-AT protein. Assuming the mass of one *E. coli* cell to be 1 pg (10^9 cells per mg of bacterial film) the number of CE6-AT proteins per cell[3,5,6] is given by:

$$\#\text{ of proteins per cell} = (\Delta/\varepsilon l)(\text{Dilution factor})/10^9$$

This method yields a value of 2.5 x 10^5 proteins per cell.

Supplementary Note 5: Calculation of CE6-AT and E6-AT copies per cell using quantitative western blotting

CE6-AT and E6-AT films of known mass were lysed in 4% SDS, 1xPBS pH 7.4 at 100 ºC for 30 min on a thermo shaker (VWR Scientific) at 900 rpm. E6-AT protein was expressed in BL21 strain and purified under denaturing conditions (8 M urea) on an Anti-His tag resin (Qiagen) and eluted with 20 mM imidazole. A BCA assay kit (Thermo Fisher) was used to measure the concentration of purified E6-AT protein in 8 M urea, Tris buffer, pH 8.0. The concentration of protein was measured to be 0.76 mg/mL. Buffer exchange for denatured, purified E6-AT into a 10 mM ammonium acetate solution was accomplished with Amicon Ultra diafiltration units (3 kDa MWCO) by repeat centrifugation and wash steps. The protein solution was mixed 1:1 with super-DHB matrix which consists of a 9:1 (w/w) mixture of 2,5-dihydroxybenzoic acid and 2-hydroxy-5-methoxybenzoic acid and analyzed by MALDI-TOF; the molecular weight was determined to be 54182. This result suggested that the pelB leader peptide was not cleaved from the protein, consistent with the observation that the expression host (BL21) cells did not aggregate in planktonic culture when induced with 0.1 mM IPTG. We then subjected purified E6-AT protein solutions of known concentration to SDS PAGE along with lysates of E6-AT and CE6-AT films. The gel was transferred to an iBlot protein transfer apparatus (Invitrogen) and the membrane was blocked with 5% milk in 0.1% tween-20 in 1x PBS for 1.5 h. Dylight 650-labeled anti-6x His tag antibody at a concentration of 0.1 µg/mL was used to stain the blot, which was imaged on a Typhoon Gel Scanner (General Electric). The mass difference caused by cleavage of the pelB leader peptide in the cell lysates is not clearly resolved on the blot. The image was analyzed by ImageJ software with the E6-AT protein used for calibration. The calibration curve is shown in **Figure S11b.** The intensities of the bands for the E6-AT and CE6-AT lysates were also quantified by ImageJ; comparison with the calibration curve allows the protein copy-number per cell to be calculated. For CE6-AT, we found $2.5x10^5 \pm 2.6x10^4$ protein copies per cell; for E6-AT $2.6x10^5 \pm 1.3x10^4$ protein copies per cell.

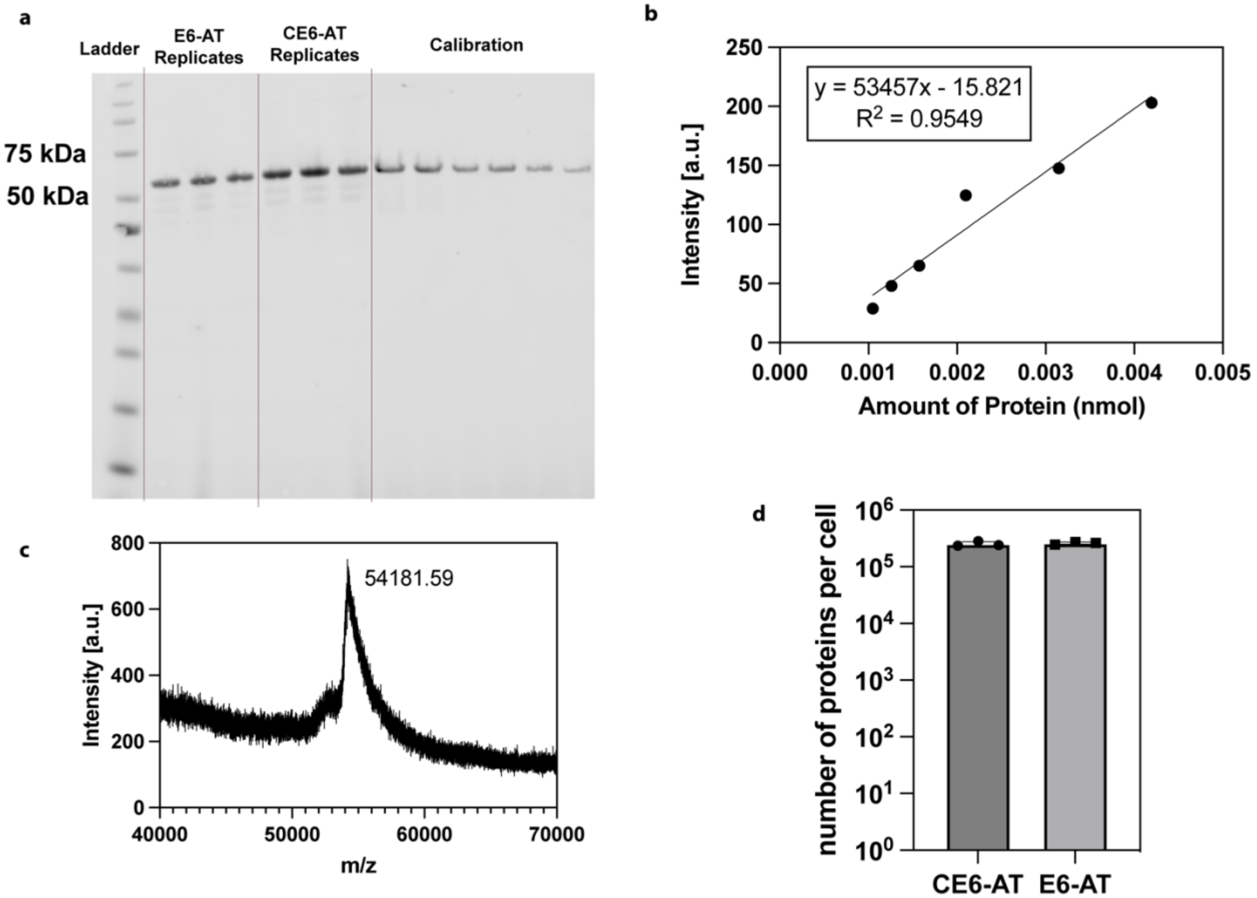

Figure S10. Quantitative western blot analysis. a, Western blot of 3 replicates of E6-AT films, 3 replicates of CE6-AT films and calibration loading of purified E6-AT at known concentrations. **b**, Calibration curve for E6-AT protein. **b**, MALDI-TOF mass spectrum of purified E6-AT. **d**, Number of proteins per cell estimated for CE6-AT and E6-AT. Both CE6-AT and E6-AT are expressed at a level of 2.5 $x10^5$ proteins per cell. Number of replicates: 3.

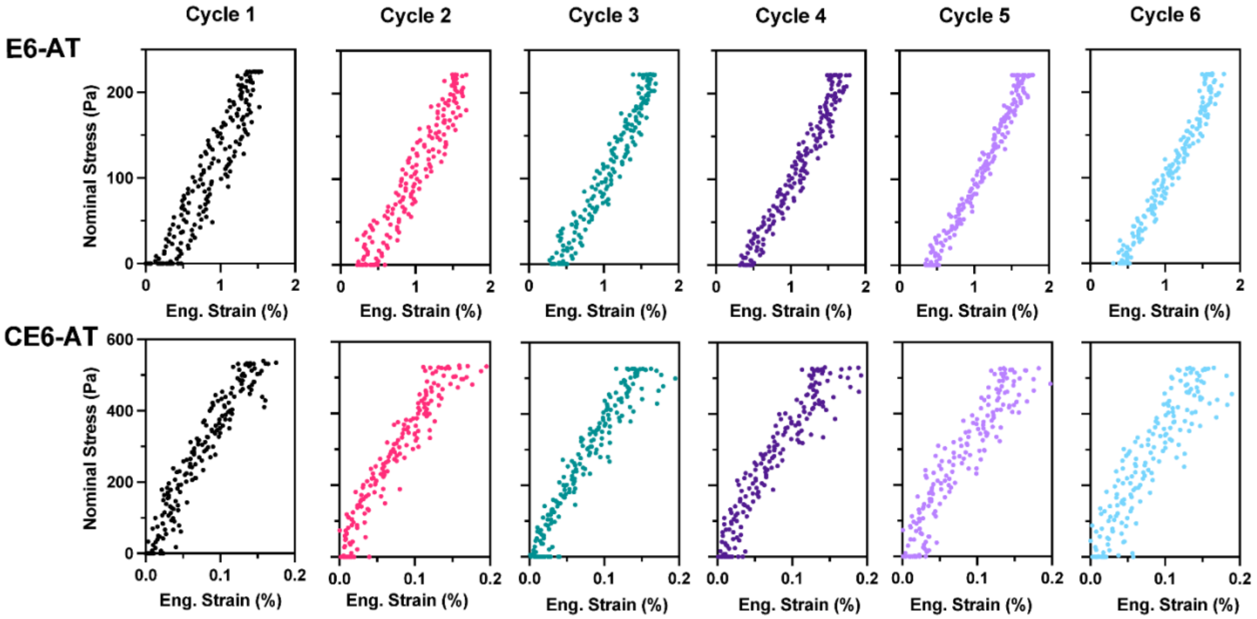

Figure S11. Stress vs strain curves for first 6 loading and unloading cycles of E6-AT and CE6-AT films in oscillatory bulge experiments. CE6 films displayed an elastic response over multiple cycles. E6 films appeared to have progressively less energy lost as hysteresis as the cycles progressed, and an initial plastic deformation in the first cycle.

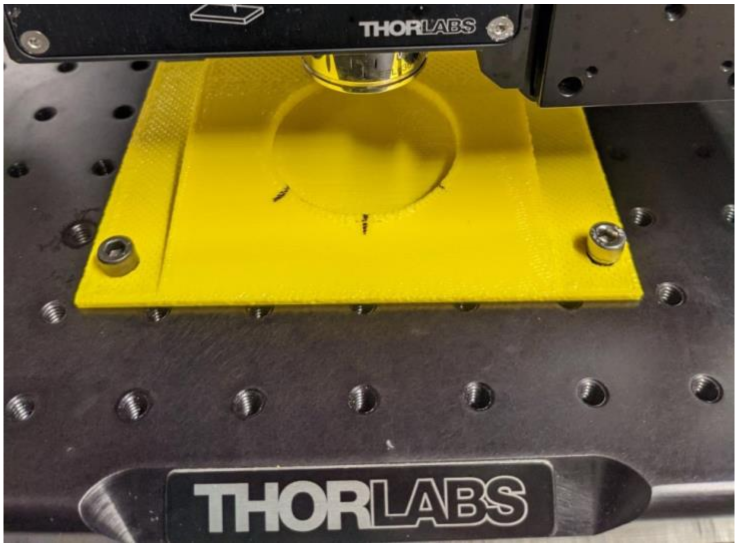

Figure S12. Sample holder for *in situ* tracking of bacterial film healing. Agar plates that held healing bacterial films were placed in the circular indent. The marks around the circular holder were aligned to similar marks on the petri dishes holding the bacterial films, and were used to ensure that the agar plate was placed in approximately the same orientation during each imaging session.

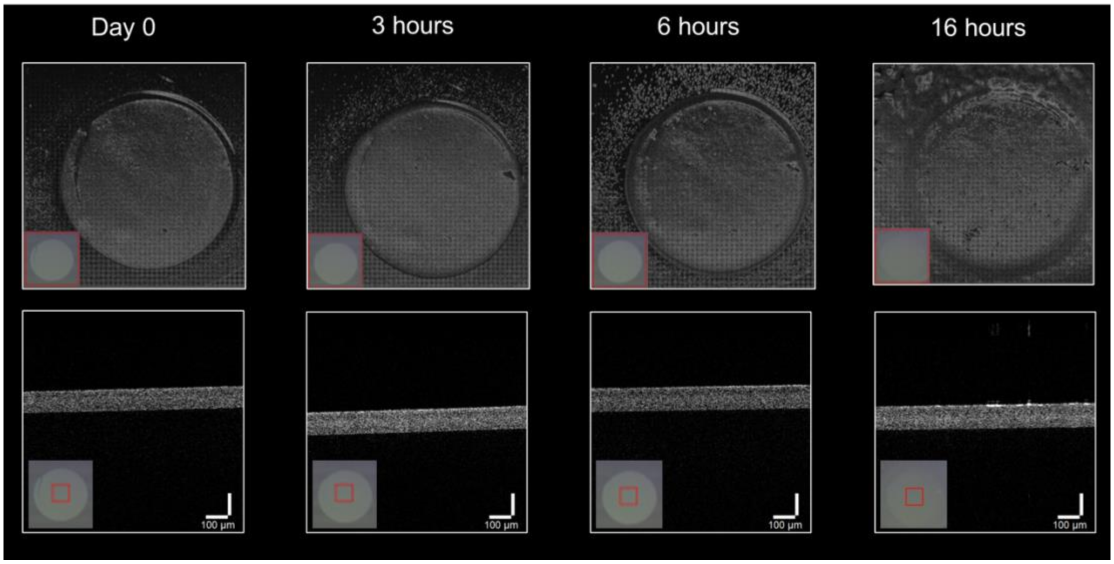

Figure S13. **OCT scans of CE6-AT control on 2YT plate**. Top row – Faint, regular patterning is due to software, not biofilm. Scale: top, 4 x 4 mm scan box; bottom, 1 x 1 x 1 mm scan box. Insets are OCT camera images, manually cropped to region being scanned. Red rectangular outline represents scan box (automatic, from ThorImage OCT software).

Table S3. Sample statistics for healed biofilms

	Total number of samples	Successfully peeled from agar and loaded (% of total)	Survived initial filling/pressure equilibration (% of total)	Failed within imposed pressures during bulge test (% of **tested**)
Original (Day 0)	4	4 (100)	4 (100)	0 (0)
Controls (16 hours)	4	4 (100)	4 (100)	2 (50)
Healed (6 hours)	4	3 (75)	0 (0)	-
Healed (12 hours)	6	5 (83)	4 (66)	4 (100)
Healed (16 hours)	7	6 (86)	3 (43)	3 (100)
Healed (24 hours)	10	8 (80)	0 (0)	-

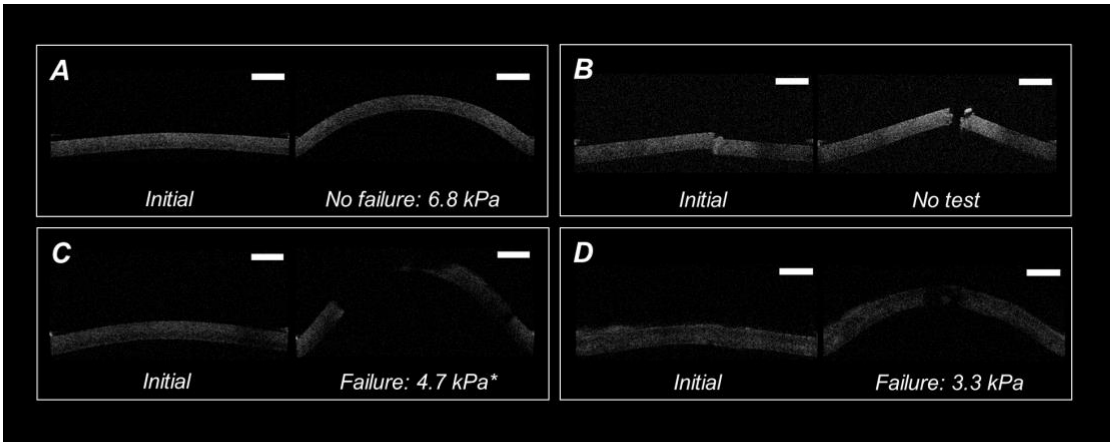

Figure S14. OCT scans of CE6-AT films with defect and healed. Original, day 7 CE6-AT films (A) do not fail within the limits of our test. Pictured maximum stress tested: 6.8 kPa. Cut films (B) immediately after injury cannot be tested, as the fluid freely flows through the tear (right). *50% of control films failed; pictured film (C) showed failure at stress: 4.7 kPa. All healed films failed (D); pictured maximum stress tolerated 3.3 kPa. Scale 200 µm.

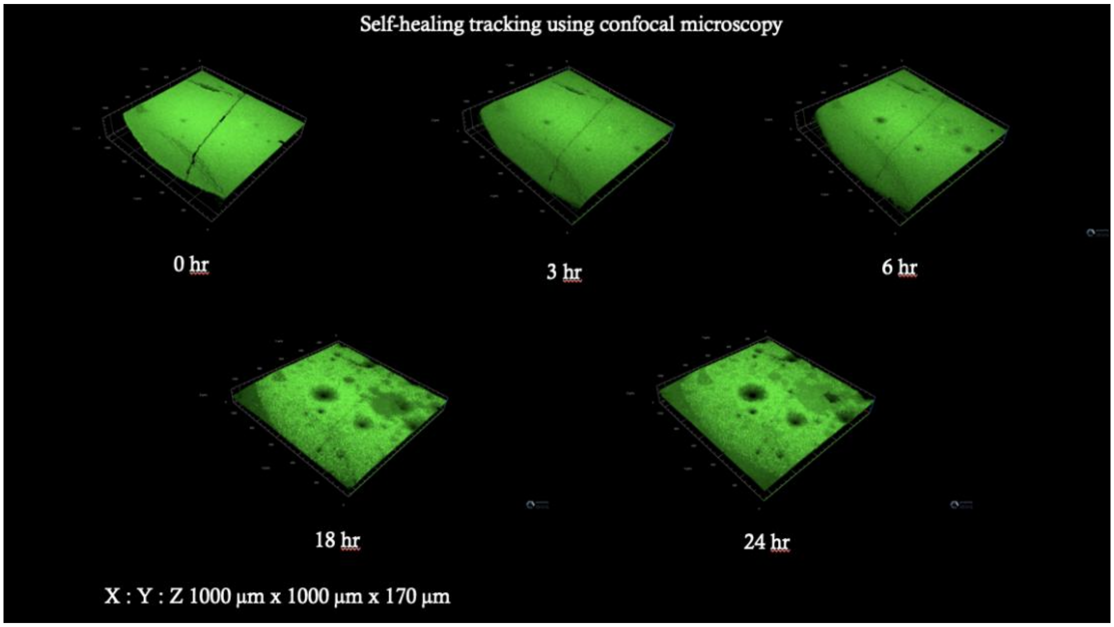

Figure S15. Confocal microscopy scanning of mWasabi CE6-AT films during healing process. Images recorded at 0, 3, 6, 18, and 24 hours. Dimensions of 3D rendering: 1000 µm x 1000 µm x 170 µm.

Supplementary Note 6. Failure of healed CE6-AT films

We assessed the extent of recovery of mechanical properties using the 16 h mark as our time point, comparing the original uncut film on day 0, the control (uninjured film grown on the healing plate), and healed films (Figure 5C-E). We observed a few clear differences among the films tested: first, the original (uninjured, day 7) films did not fail at the maximum pressure imposed by our device. The actual maximum stress depended on the sample thickness and properties, but the highest observed stress tested on a CE6-AT sample of 89 µm thickness was 7.21 kPa. While there was some variation in the range of applied stresses and observed strains during our test, we observed that CE6-AT films on day 7 consistently tolerated stresses greater than 6.5 kPa (Figure S14A). In contrast, two out of four of the control films (16 h) failed during the experiment. One failed at a stress of 4.7 kPa (Figure S14C), while the other film failed at a stress of 3.3 kPa (Figure S14D). All of the healed films that could be loaded and tested failed within the range of stresses applied in the test. To probe the extent of healing of the defect, we used CE6-AT films expressing the fluorescent protein mWasabi, and observed the healing process using confocal microscopy (which has higher resolution than OCT). Confocal microscopy images (Figure S15) revealed visible defects until 16 h of healing.

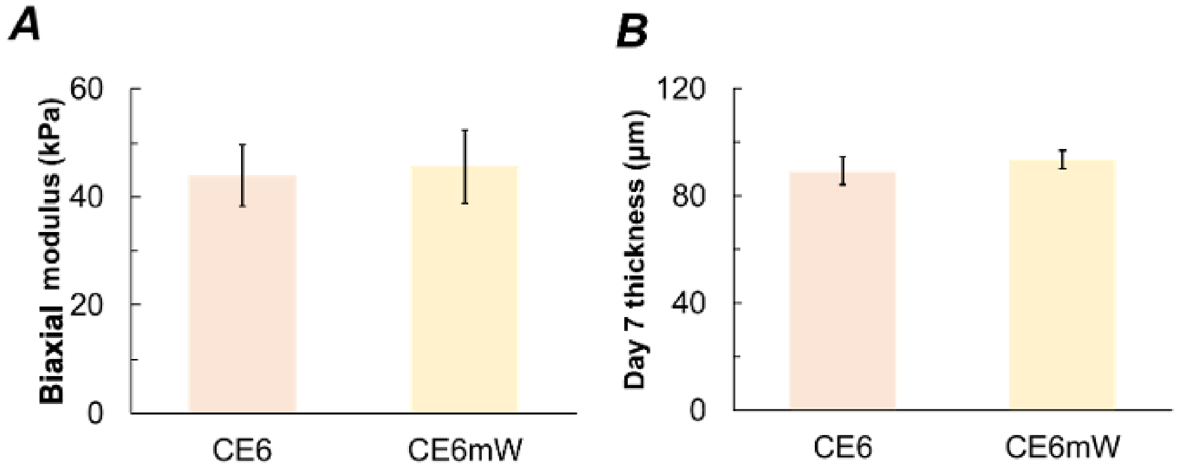

Figure S16. CE6mW (CE6 mWasabi) exhibited modulus (A) and day 7 thickness (B) similar to those of CE6. Modulus: 44.0 ± 5.63 kPa (CE6), 45.6 ± 6.7 kPa (CE6mW). Thickness: 89.4 ± 5.07 µm (CE6), 93.6 ± 3.53 µm (CE6mW). Day 7 films did not fail within the pressure range imposed by our test.

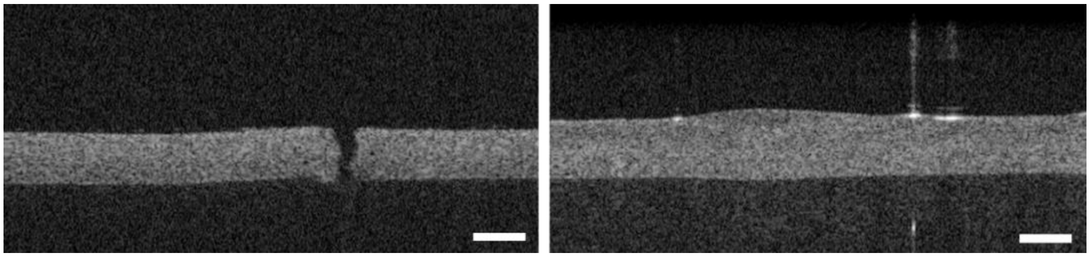

Figure S17. E6mC (E6 mCherry) can also heal after injury. OCT images taken immediately (left) and after 16 h (right) show growth of biofilm in previously cut region. See main text for protocols. Scale bar 100 μm.

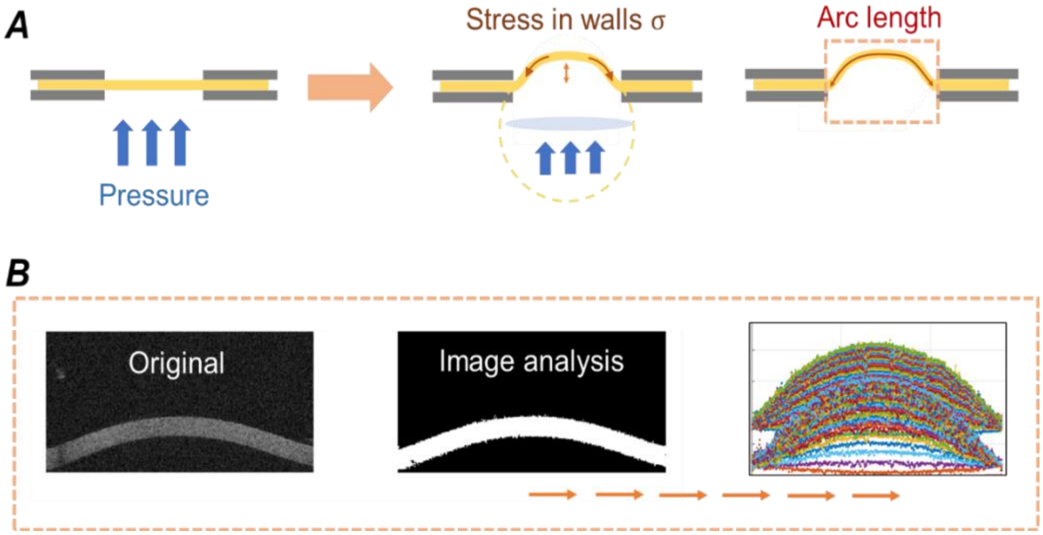

Figure S18. (A) Spherical cap approximation for stress assumes that deformed film is part of a larger spherical pressure vessel and the stress in the walls of the film balances the applied pressure. Strain is estimated as a difference in the arc length (red arrow) compared to the original (flat) length of the biofilm. (B) Image processing scripts binarize and clean up OCT images and detect the top and bottom surfaces of the film over thousands of images (bottom right).

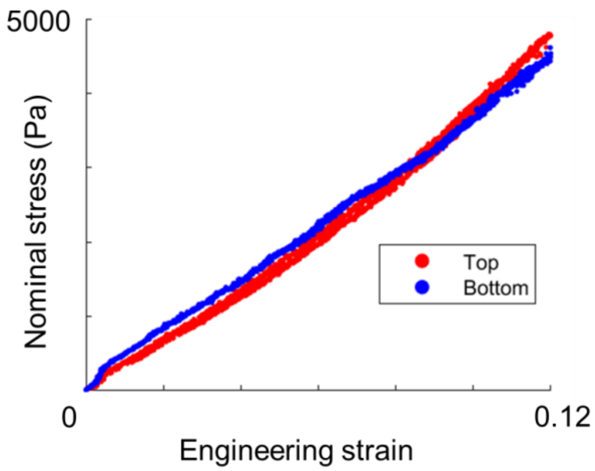

Figure S19. We observed minimal discrepancy between stress-strain curves from the top vs. bottom surfaces of the bacterial film.

References

(1) Kozlowski, M. T.; Silverman, B. R.; Johnstone, C. P.; Tirrell, D. A. Genetically Programmable Microbial Assembly. *ACS Synth. Biol.* **2021**, *10* (6), 1351–1359.

(2) HEPES Buffer. *Cold Spring Harb Protoc* **2010**, *2010* (7).

(3) Kamihira, M.; Taniguchi, M.; Kobayashi, T. Sterilization of Microorganisms with Supercritical Carbon Dioxide. *Agri Biol Chem* **1987**, *51* (2), 407–412.

(4) *The Beer-Lambert Law*. Chemistry LibreTexts. https://chem.libretexts.org/Bookshelves/Physical_and_Theoretical_Chemistry_Textbook_Maps/Supplemental_Modules_(Physical_and_Theoretical_Chemistry)/Spectroscopy/Electronic_Spectroscopy/Electronic_Spectroscopy_Basics/The_Beer-Lambert_Law (accessed 2023-08-05).

(5) Cayley, S.; Lewis, B. A.; Guttman, H. J.; Record, M. T. Characterization of the Cytoplasm of *Escherichia coli* K-12 as a Function of External Osmolarity. Implications for Protein-DNA Interactions in Vivo. *J Mol Biol* **1991**, *222* (2), 281–300.

(6) Brunschede, H.; Dove, T. L.; Bremer, H. Establishment of Exponential Growth after a Nutritional Shift-up in *Escherichia coli* B/r: Accumulation of Deoxyribonucleic Acid, Ribonucleic Acid, and Protein. *J Bacteriol* **1977**, *129* (2), 1020–1033.
